# Mechanistic studies of an L-proline-catalyzed pyridazine formation involving a Diels–Alder reaction with inverse electron demand

**DOI:** 10.3762/bjoc.15.3

**Published:** 2019-01-03

**Authors:** Anne Schnell, J Alexander Willms, S Nozinovic, Marianne Engeser

**Affiliations:** 1 University of Bonn, Kekulé-Institute of Organic Chemistry and Biochemistry, Gerhard-Domagk-Str. 1, D-53121 Bonn, Germany

**Keywords:** charge-tag, electrospray ionization, enamine organocatalysis, L-proline, reaction mechanism

## Abstract

The mechanism of an L-proline-catalyzed pyridazine formation from acetone and aryl-substituted tetrazines via a Diels–Alder reaction with inverse electron demand has been studied with NMR and with electrospray ionization mass spectrometry. A catalytic cycle with three intermediates has been proposed. An enamine derived from L-proline and acetone acts as an electron-rich dienophile in a [4 + 2] cycloaddition with the electron-poor tetrazine forming a tetraazabicyclo[2.2.2]octadiene derivative which then eliminates N_2_ in a retro-Diels–Alder reaction to yield a 4,5-dihydropyridazine species. The reaction was studied in three variants: unmodified, with a charge-tagged substrate, and with a charge-tagged proline catalyst. The charge-tagging technique strongly increases the ESI response of the respective species and therefore enables to capture otherwise undetected reaction components. With the first two reaction variants, only small intensities of intermediates were found, but the temporal progress of reactants and products could be monitored very well. In experiments with the charge-tagged L-proline-derived catalyst, all three intermediates of the proposed catalytic cycle were detected and characterized by collision-induced dissociation (CID) experiments. Some of the CID pathways of intermediates mimic single steps of the proposed catalytic cycle in the gas phase. Thus, the charge-tagged catalyst proved one more time its superior effectiveness for the detection and study of reactive intermediates at low concentrations.

## Introduction

**Figure 1:**
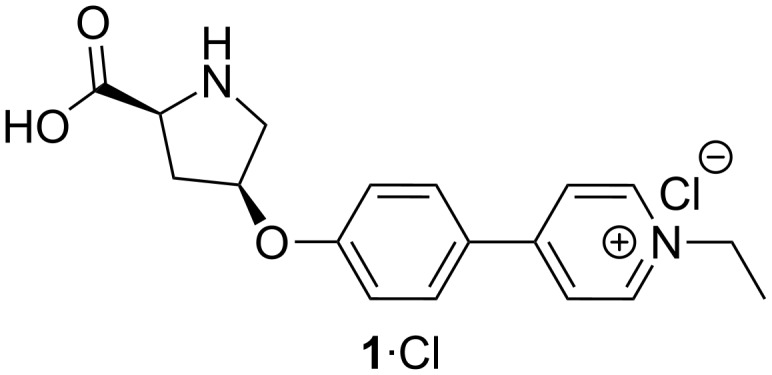
Charge-tagged L-proline-derived catalyst **1**∙Cl [18].

Electrospray (ESI) mass spectrometry (MS) [1] is well suited for studying reaction mechanisms as it is a soft ionization method leaving most species intact [1–3]. In addition, it is a fast analytical method [3] making it possible to study transient intermediates [4–6]. Various types of reactions have been studied successfully by ESIMS ranging from Ziegler–Natta polymerization [7] and coupling reactions [8–9] to organic reactions such as the Baylis–Hillman [10–15], aldol [16–18] or Diels–Alder reactions [19–20]. An advantageous feature of high-resolution ESIMS is that each ionic species in the gas phase produces distinct signals which are unlikely to be overlaid with signals from other species [6]. As a consequence, reaction mixtures typically containing many different species can be analyzed without prior separation of the components [5–6]. As a drawback of MS, isomers typically are hard to analyze as they have the same mass and thus lead to the same signal. However, they can be distinguished in fortunate cases by more sophisticated approaches like tandem mass spectrometry [3], ion mobility mass spectrometry [21], or coupling with liquid or gas chromatography. Further, ESI signal intensities do not directly correlate to concentrations in solution, but to the ESI response of the pertaining molecules [3,22]. The ESI response is influenced by a variety of factors like chargeability and surface activity of a given analyte and also by the applied electrospray conditions [22]. If there are big differences in the ESI response between species of interest, some species might dominate the spectrum so much that species with a low ESI response are concealed [2,17]. To counteract these problems, covalently linked charge-tags can be introduced into the analyte molecules, usually in the form of alkylated amines or phosphines [5–6]. The charge-tag can be located either within the substrate [23–25] or the catalyst [18,26–27]. As a result, all species containing the charge-tag will have a similarly high ESI response [6,25] while species that are not involved in the reaction and do not carry the charge-tag will have a much lower ESI response. A charge-tag thus facilitates “fishing” [5,23,28] for reactive intermediates. We have previously used the charge-tagged L-proline derived catalyst **1**∙Cl (Figure 1) in an ESIMS study of a L-proline-catalyzed aldol reaction.

Organocatalysis has become a major research field with many applications and has proven to be a valuable complementary approach to organometallic or enzymatic catalysis [29–34]. The advantages especially in comparison to organometallic catalysis lie in a lower toxicity, air sensitivity and lower costs [34]. A huge repertoire of organocatalyzed reactions have been published in recent years with high efficiencies and selectivities [29,33,35–39]. Proline as a natural amino acid is a perfect example of an organocatalyst. Both enantiomers are inexpensive and easily available. The work of List and Barbas in 2000 was groundbreaking for L-proline-catalyzed reactions [40]. They published a L*-*proline-catalyzed asymmetric aldol reaction and suggested that the essential catalytic step is the enamine formation between the secondary amine function of L-proline and the carbonyl substrate acetone [40]. Houk and co-worker [41] verified the mechanism with quantum mechanical calculations, thus giving rise to the “List–Houk*”* mechanism. A discussion about the role of oxazolidinones as isomeric species to enamines has been raised in the scientific community [42–47]. Tetrazines and their reactivity in Diels–Alder reactions with inverse electron demand are of interest in the field of biology [48–49]. Very recently, they have been studied by mass spectrometric means [50].

**Scheme 1:**
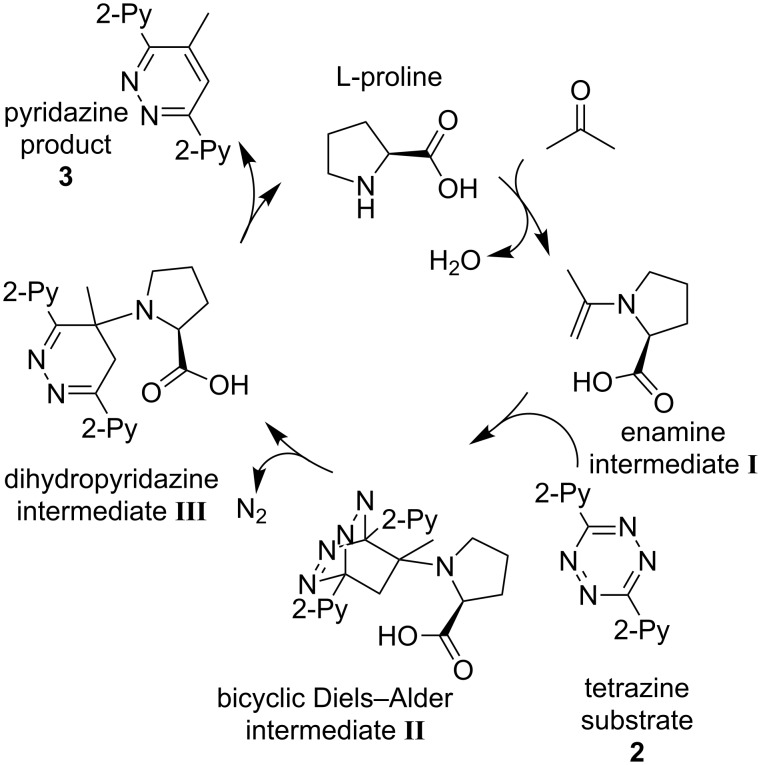
Putative catalytic cycle [51] for the L-proline-catalyzed Diels–Alder reaction with inverse electron demand.

In 2008, Xie et al. [51] published an L-proline-catalyzed reaction between ketones and aryl-substituted 1,2,4,5-tetrazines which leads to functionalized pyridazines. They also postulated a mechanism (Scheme 1) for the reaction [51]. Based on the knowledge that secondary amines catalyze the formation of enamines from ketones and other carbonyl compounds [33], an initial formation of the enamine **I** seems plausible. It is an electron-rich dienophile which could undergo a [4 + 2] cycloaddition with the electron-poor aryl-substituted tetrazine **2** in a Diels–Alder reaction with inverse electron demand. The bicyclic Diels–Alder intermediate **II** then might undergo a retro-Diels–Alder reaction by eliminating dinitrogen. This leads to the dihydropyridazine intermediate **III** out of which the catalyst is released to yield the pyridazine product **3** [51].

Shihab et al. later studied a related reaction of a series of dienophiles with dimethyl 1,2,4,5-tetrazine-3,6-dicarboxylate by theoretical methods [52]. Their results are in agreement with the catalytic cycle presented in Scheme 1. However, the question is raised whether the bicyclic Diels–Alder species **II** is a real intermediate or rather a transition state of a concerted formation of the dihydropyridazine intermediate **III** directly from the enamine/dienophile **I** and the tetrazine. Thus, we decided to use NMR (nuclear magnetic resonance) spectroscopy and ESI mass spectrometry in combination with a charge-tagging strategy to get deeper insights in the presence or absence of the three intermediates by experimental means.

## Results and Discussion

### Synthesis

**Scheme 2:**
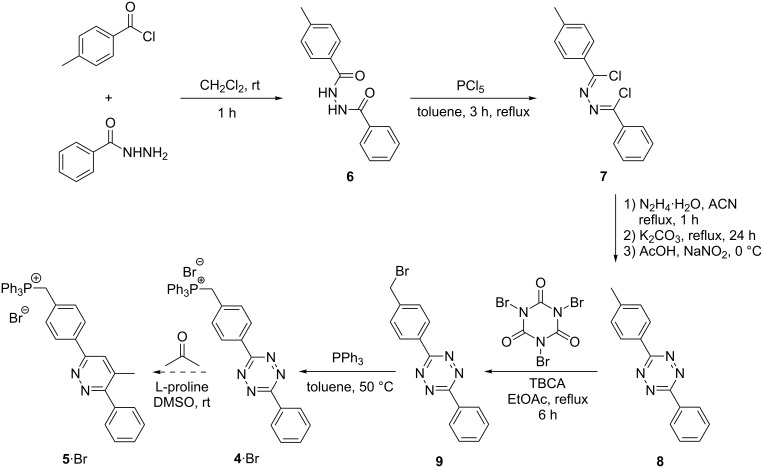
Synthesis of the charge-tagged tetrazine **4**∙Br as a reactant for the proline-catalyzed Diels–Alder reaction leading to **5**∙Br.

In addition to the charge-tagged proline catalyst **1**∙Cl [18], the charge-tagged tetrazine substrate **4**∙Br was synthesized (Scheme 2). We were inspired by the work of McIndoe and co-workers [6,25,53] who introduced and established the triphenylphosphonium charge-tag. The corresponding Diels–Alder reaction starting from this reactant yields pyridazine product **5**∙Br. The first two synthetic steps (Scheme 2) towards benzenehydrazonoyl chloride **7** [54] were performed according to the protocol of Wang et al. [55]. The formation of new tetrazine compound **8** was performed in accordance to the procedure published by Liu et al. [56] and bromination to the new benzyl bromide **9** succeeded with tribromoisocyanuric acid (TBCA) as published by de Almeida et al. [57]. The final transformation of benzyl bromide **9** to triphenylphosphonium charge-tagged **4**∙Br was performed with a slightly modified protocol from Vikse et al. [53].

### Mechanistic studies: ^1^H NMR experiments

**Scheme 3:**
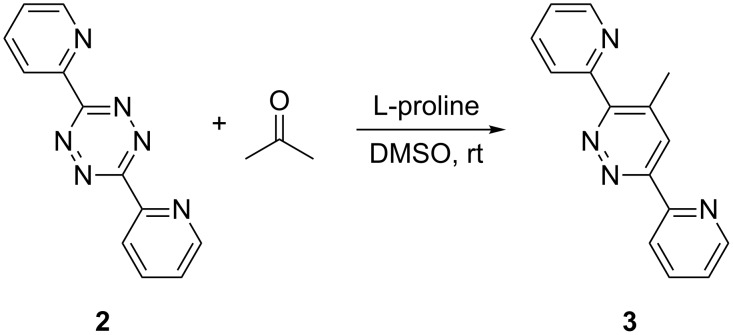
Reaction R1: L-proline-catalyzed reaction between **2** and acetone.

**Figure 2:**
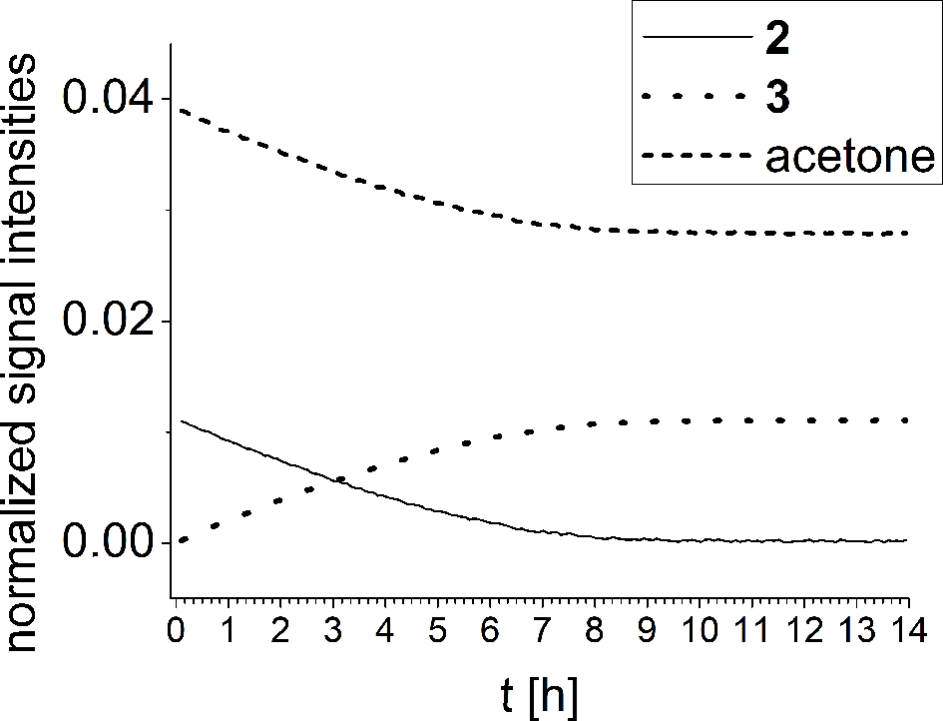
NMR monitoring of reaction R1 in deuterated DMSO (concentration of tetrazine 0.005 mmol/mL).

^1^H NMR experiments of reaction R1 (Scheme 3) show the temporal progress of the reaction which is easily tracked by the concentrations of substrate **2**, acetone and product **3** (Figure 2, and Figures S1 and S2 in Supporting Information File 1). However, no reaction intermediates could be detected either in this case or with enhanced concentrations (Figure S3, Supporting Information File 1).

**Scheme 4:**
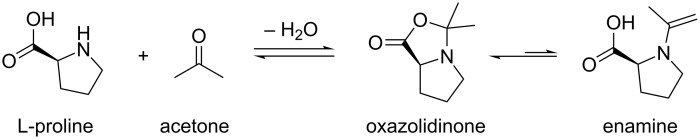
Equilibrium of oxazolidinone and enamine formation.

In absence of tetrazine **2**, the ^1^H NMR spectra of a reaction mixture only containing L-proline and acetone in deuterated DMSO show an additional small signal at δ = 4.4 ppm (Figure S3, Supporting Information File 1), characteristic for an oxazolidinone (Scheme 4). In agreement with the findings from List [58] and Gschwind [46], the equilibrium concentration of the isomeric enamine is too low to be detected although this species is required as dienophile for the Diels–Alder reaction to proceed.

### Mechanistic studies: ESIMS experiments

**Figure 3:**
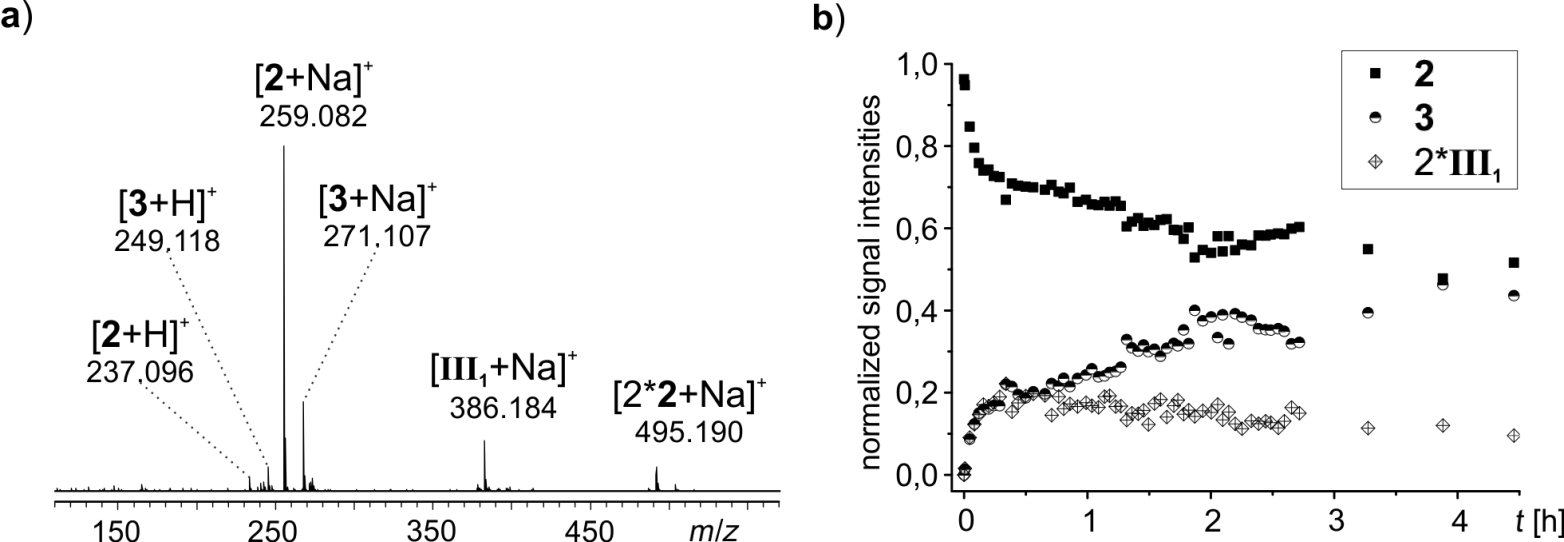
a) ESI mass spectrum of reaction R1 after 26 min. b) ESIMS monitoring of reaction R1. To better visualize the trend of **III_1_**, the signal intensities of **III_1_** have been multiplied by a factor of two.

As ESIMS has a lower limit of detection than NMR, we also studied the proceeding reaction with ESIMS. In its simplest version, i.e., without charge-tagged components (R1, Scheme 3) and at a low concentration (0.005 mmol/mL of tetrazine), the temporal progress of substrate **2** and product **3** could be followed directly (Figure S5, Supporting Information File 1). Unfortunately, no reaction intermediates were detected. Using a lower amount of solvent (0.2 mmol of tetrazine in 10 mL of dimethyl sulfoxide) has the downside that not all of the substrate **2** gets dissolved initially. Product **3** is completely soluble at this concentration, so only while the substrate **2** transforms into product **3**, **2** gets fully dissolved. Small samples were taken from the reaction flask at regular intervals, diluted and immediately analyzed by ESIMS. The decay of substrate **2** (*m*/*z* 237, *m*/*z* 259, *m*/*z* 495) and the increase of product **3** (*m*/*z* 249, *m*/*z* 271) were easily observed (Figure 3a,b). Furthermore, the dihydropyridazine intermediate **III_1 _***(m*/*z* 386) could be detected for the first time (Figure 3a). The intensity of **III_1_** initially rises more quickly than the intensity of product **3** and declines very slowly as the reaction progresses (Figure 3b). However, no signals for the intermediates **I_1_** (enamine) and **II_1_** (bicyclic Diels–Alder intermediate) were found.

**Figure 4:**
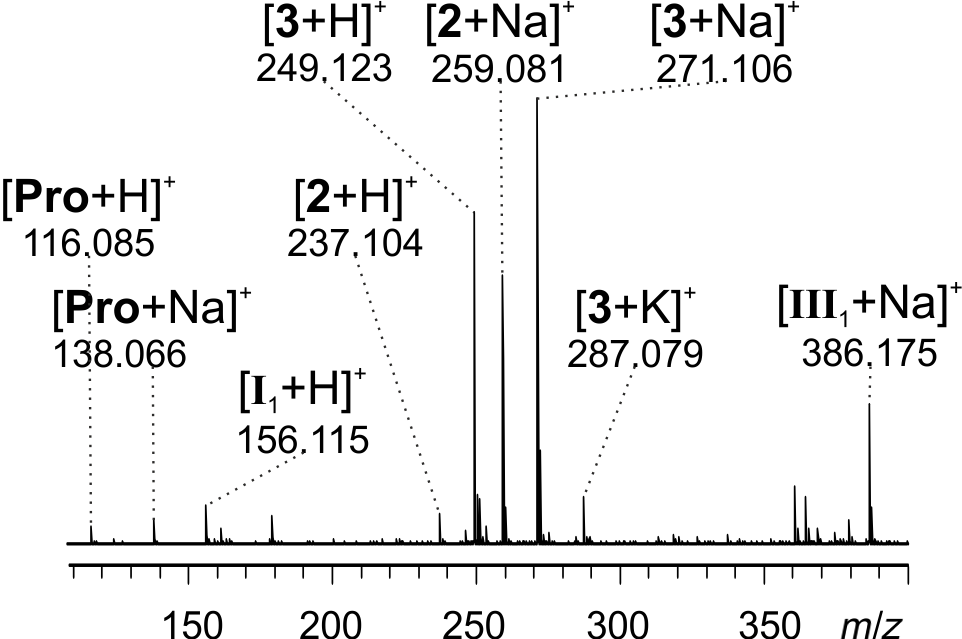
ESI mass spectrum of reaction R1 with preformed **I_1_** 8 minutes after adding substrate **2**.

Intermediate **I_1_** (*m*/*z* 156) has been observed before by Marquez et al. in ESIMS experiments of an aldol reaction [17]. In our case, it unfortunately does not accumulate in sufficient amounts for detection. Thus, the reaction was setup differently: instead of premixing L-proline (0.05 equiv) and tetrazine substrate **2** (1 equiv) in solution and then adding acetone (4 equiv) to start the reaction as before, now L-proline (1 equiv) and acetone (95 equiv) were mixed first. The formation of intermediate enamine **I_1_** and/or the isomeric oxazolidinone was validated by the detection of a signal at *m*/*z* 156 for the protonated species in ESIMS spectra, and only then the tetrazine substrate **2** (1 equiv) was added. By this way, it was possible to detect not only substrate **2** (*m*/*z* 237, *m*/*z* 259), product **3** (*m*/*z* 249, *m*/*z* 271, *m*/*z* 287) and the proline catalyst (*m*/*z* 116, *m*/*z* 138), but also the intermediate **I_1_** and/or its oxazolidinone isomer (*m*/*z* 156) and the dihydropyridazine intermediate **III_1_** (*m*/*z* 386, Figure 4) in the reacting solution.

**Scheme 5:**
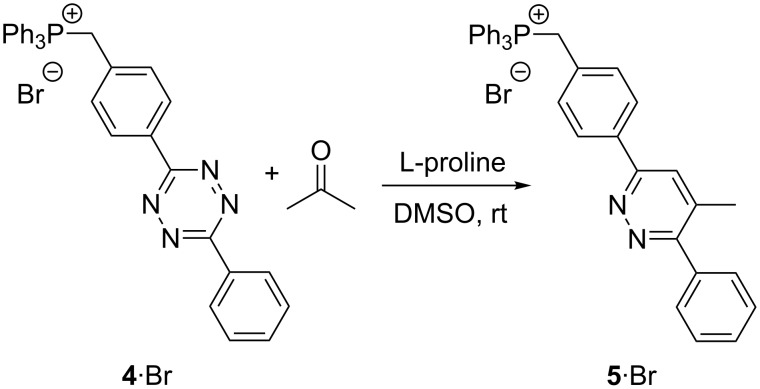
Reaction R2: L-proline-catalyzed reaction between charge-tagged substrate **4**∙Br and acetone. The regioselectivity has not been specified. **5**∙Br could be either regioisomer (Scheme S1, Supporting Information File 1).

In order to enhance the ESI response of putative reactive intermediates, the reaction was performed with the charge-tagged tetrazine **4**∙Br (R2, Scheme 5).

**Figure 5:**
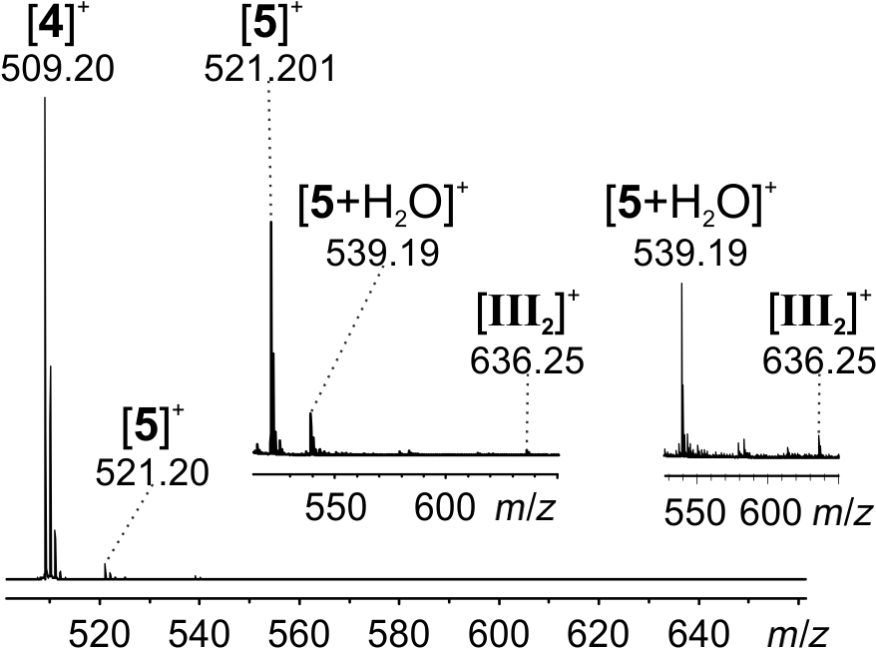
ESI mass spectrum of reaction R2 using a continuous-flow setup with a calculated reaction time of 86 s. The two insets show zooms into relevant parts of the spectrum.

A continuous-flow setup [4,17–18] was used for fast sampling of the reaction R2 directly after its initiation. A solution of substrate **4**∙Br and L-proline was mixed with acetone in a commercial PEEK microreactor mixing tee. The reacting solution was diluted with acetonitrile using a second microreactor and subsequently fed into the ESI source of the mass spectrometer. Beside a very prominent signal of the charge-tagged substrate **4** (*m*/*z* 509), signals corresponding to the product **5** (*m*/*z* 521, *m*/*z* 539) were observed (Figure 5). In addition, a low intensity signal for the dihydropyridazine intermediate **III_2_** (*m*/*z* 636) could be found. The enamine intermediate **I_2_** does not carry a charge-tag in this experiment and thus was not detected. Unfortunately, any indications for the charge-tagged bicyclic Diels–Alder intermediate **II_2_** could not be found either.

**Figure 6:**
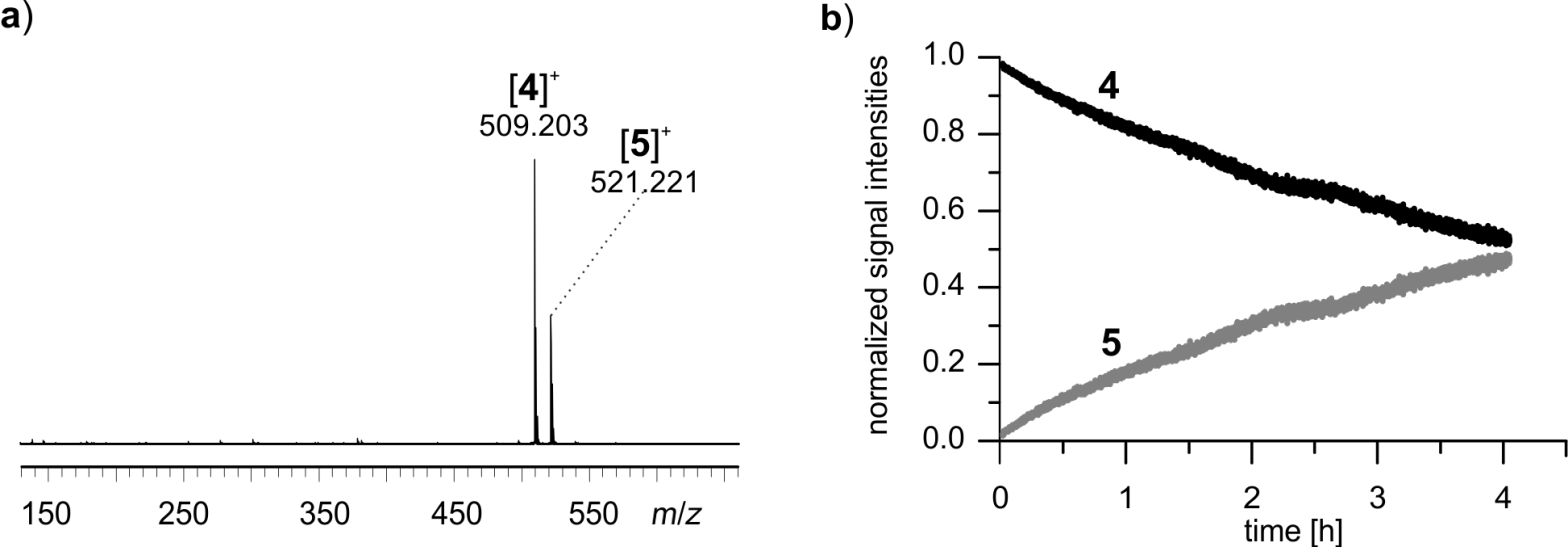
a) Reaction R2 after two hours (syringe setup). b) ESIMS monitoring of reaction R2. Signal intensities for substrate **4** and product **5** are depicted.

To study the reaction R2 over a longer period of time, substrate **4**∙Br, acetone and L-proline were simply mixed in a syringe and directly fed into the ESI mass spectrometer over a time span of 4 hours. The signals for substrate **4** (*m*/*z* 509) and product **5** (*m*/*z* 521, Figure 6a) were detected. Approximately 50% conversion was achieved after 4 hours at room temperature (Figure 6b). No signals corresponding to the bicyclic Diels–Alder intermediate **II_2_** and the dihydropyridazine intermediate **III_2_** could be detected, even though the charge-tagging strategy should have facilitated their detection. Clearly, the presence of high amounts of **4**∙Br and **5**∙Br suppressed ESI signals of other species of interest.

**Scheme 6:**
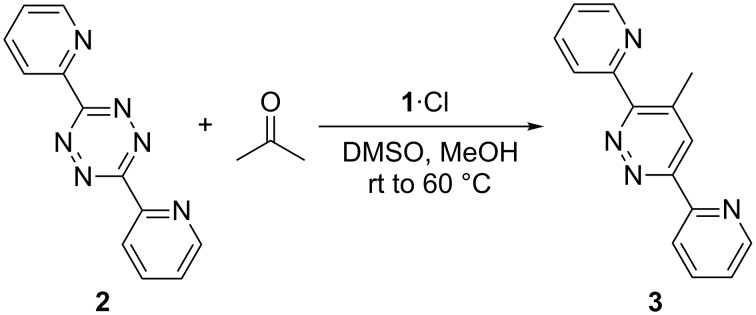
Reaction R3: substrate **2**, acetone and charge-tagged catalyst **1**∙Cl.

In contrast, less equivalents of charge-tagged species are present in the reaction solution if the charge-tag is part of the catalyst. The intermediates might not be concealed under these conditions. Therefore, substrate **2** and acetone were mixed with the charge-tagged catalyst **1**∙Cl in a third variant of the reaction (R3, Scheme 6).

**Figure 7:**
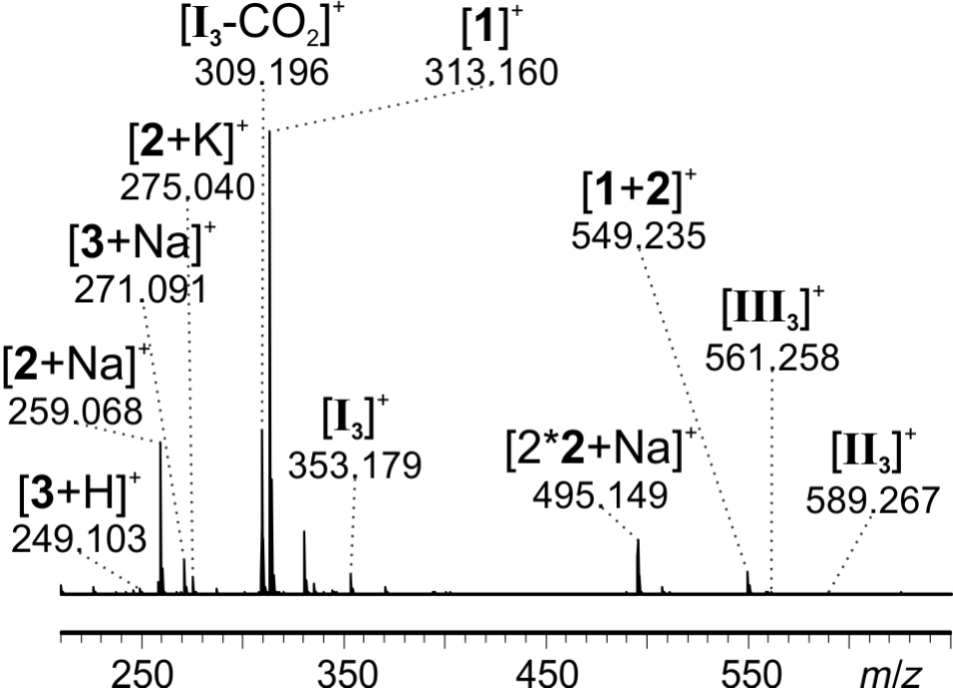
ESI mass spectrum of reaction R3 at 60 °C after 1.5 h.

As the reaction R3 does not show conversion at room temperature, the mixture was successively heated up to 60 °C. Small samples were taken from the reaction flask at regular intervals, diluted and fed into the ESI source. Signals corresponding to tetrazine substrate **2** (*m*/*z* 259, *m*/*z* 275, *m*/*z* 495, *m*/*z* 549), pyridazine product **3** (*m*/*z* 249, *m*/*z* 271) and catalyst **1** (*m*/*z* 313, *m*/*z* 549) were observed as expected (Figure 7).

**Scheme 7:**
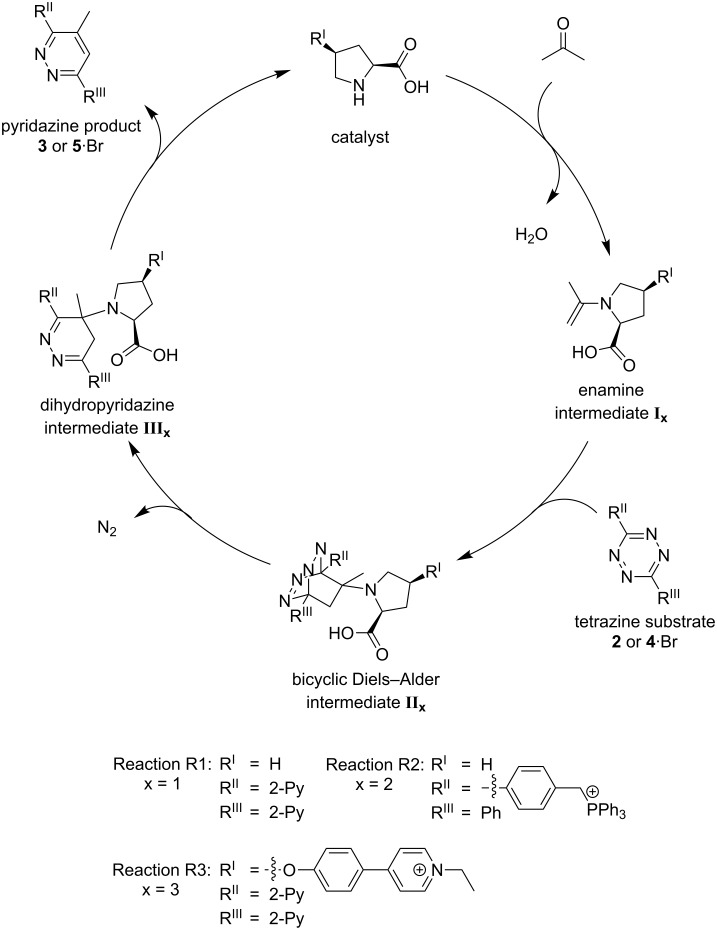
General catalytic cycle for reactions R1–R3.

In addition, signals corresponding to all three proposed intermediates (Scheme 7) were found: intermediate **I_3_** (*m*/*z* 353), dihydropyridazine intermediate **III_3_** (*m*/*z* 561) and, for the first time, the most intriguing bicyclic Diels–Alder intermediate **II_3_** (*m*/*z* 589). It has to be emphasized that each of these species was detected as an unmodified ion. As all intermediates are formed by reaction with the charge-tagged catalyst, they are inherently charged and do not need to be protonated during the ionization process. Ion **I_3_** very easily loses CO_2_ which causes the signal at *m*/*z* 309. This behavior could be confirmed by induced fragmentation experiments (see below).

**Figure 8:**
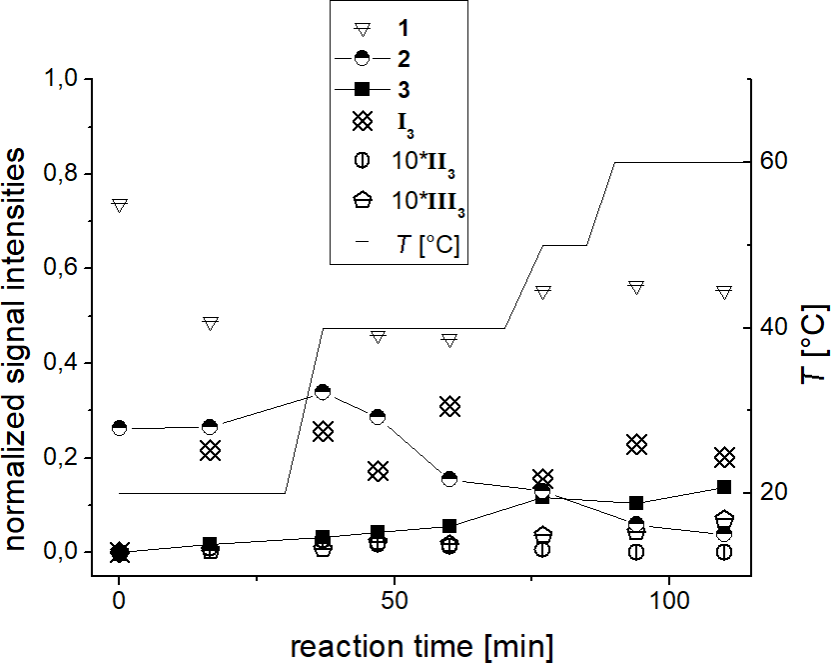
ESIMS monitoring of reaction R3. The plotted intensity values for each molecule are a sum of all corresponding signal intensities (i.e., [M + H]^+^, [M + Na]^+^, etc.). Signal intensities of **II_3_** and **III_3_** have been multiplied by a factor of ten for better visualization.

Monitoring the temporal progress of reaction R3 was achieved by taking small samples at regular intervals, diluting and swiftly feeding them into the spectrometer (Figure 8). The conversion of substrate **2** to product **3** can easily be followed by the change of the respective signal intensities. The signal intensities for the intermediates stay relatively constant and are rather low for **II_3_** and **III_3_**. The relatively high signal intensity of the intermediate **I_3_** in contrast to the other two intermediates might indicate that the [4 + 2] cycloaddition between enamine intermediate **I_3_** and tetrazine substrate **2** in R3 does not proceed as easily as for the untagged reaction R1 discussed above. This difference in the kinetic behavior might be due to the higher steric hindrance for the [4 + 2] cycloaddition with the charge-tagged enamine intermediate **I_3_** in comparison to the untagged enamine intermediate **I_1_**. Thus, the use of the charge-tagged catalyst was essential for the detection of the elusive, but mechanistically most interesting intermediate **II_3_**. However, this comes at the cost of putative changes of both the overall energy barrier of the reaction (R3 is significantly slower than R1) as well as the relative energetics of the elementary steps in the catalytic cycle (visible in the abundance ratio of intermediates).

All three intermediates could be further characterized by collision induced dissociation (CID) experiments (see below).

**Figure 9:**
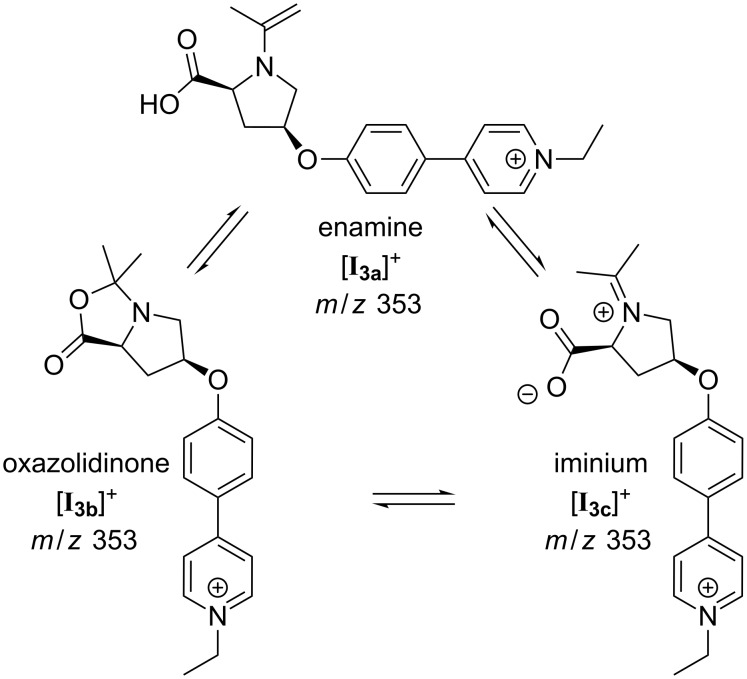
Isomeric forms in equilibrium: enamine [**I_3a_**]^+^, oxazolidinone [**I_3b_**]^+^ and iminium [**I_3c_**]^+^.

**Figure 10:**
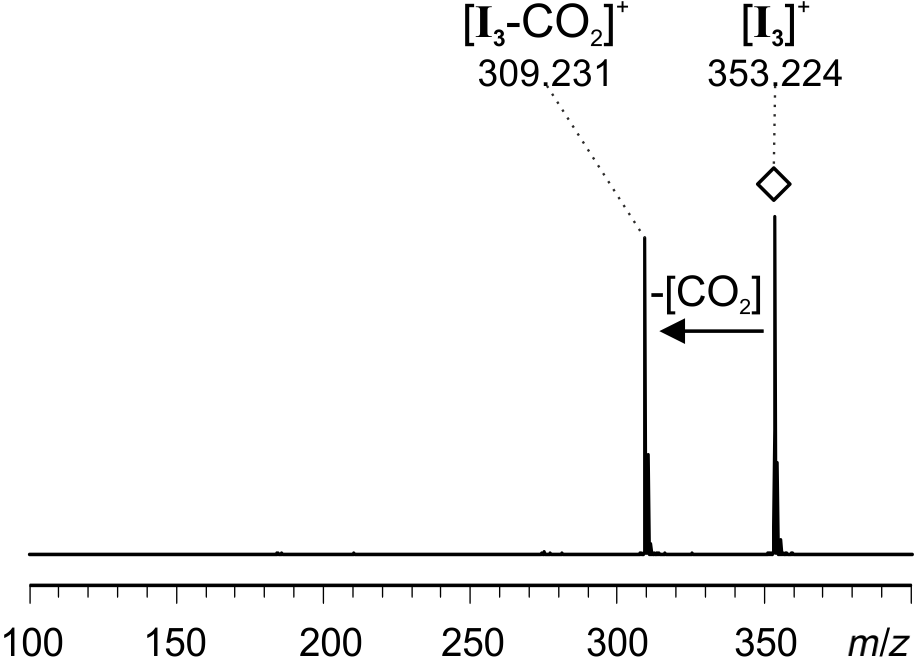
ESI(+) CID spectrum of mass-selected [**I_3_**]^+^ (*m*/*z* 353); collision energy voltage 1 V.

The signal at *m*/*z* 353 of intermediate **I_3_** can correspond to three isomeric forms, i.e., enamine [**I_3a_**]^+^, oxazolidinone [**I_3b_**]^+^ or iminium [**I_3c_**]^+^ (Figure 9). The oxazolidinone species is well known to exist in reacting solutions of L-proline-catalyzed reactions [43,45–47], but the enamine species has been detected as well [46]. The equilibrium is highly solvent-dependent. Gschwind and co-workers [46] found that for the condensation of L-proline with propanal in DMSO, 9% of the resulting species are the enamine species and 91% the two possible diastereomeric oxazolidinone species, and the oxazolidinone is the only NMR-detectable species with acetone in DMSO. As only the enamine species can act as a dienophile and not the oxazolidinone species, we here expect the presence of both isomers in the reacting solution with the oxazolidinone present in large excess. Upon CID of the mass-selected signal for **I_3_** at *m*/*z* 353 (Figure 10), only elimination of CO_2_ was observed. The spectra very much resemble the ones we obtained for ions with the same *m*/*z* observed when spraying acetonitrile solutions of **1**∙Cl and acetone. These were characterized as the oxazolidinone species [**I_3b_**]^+^ by infrared multiphoton dissociation (IRMPD) action spectroscopy in the gas phase [18,59]. The fragmentation already takes place at very low collision energies, so that some amount of fragmentation is expected to occur in the ESI source under normal ESI conditions – in accordance with the experimental observation as depicted in Figure 7.

**Figure 11:**
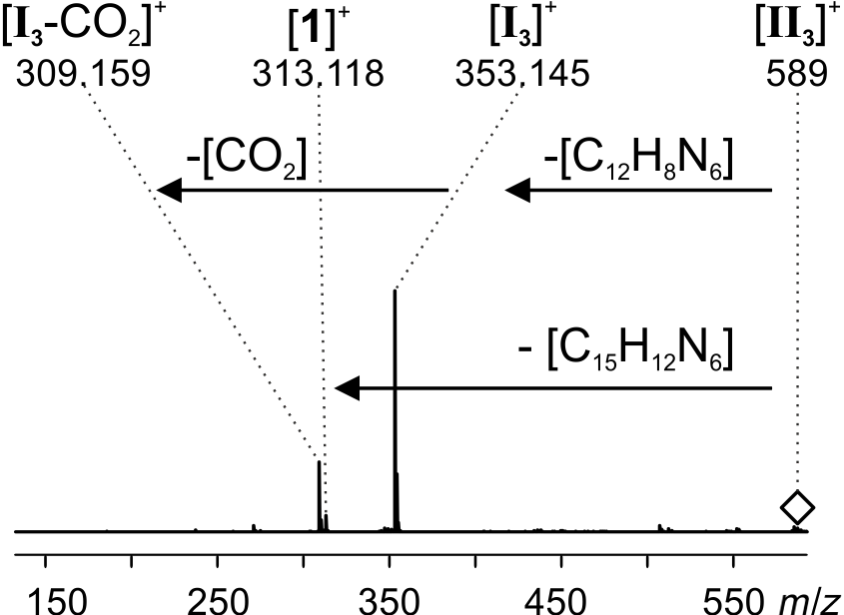
ESI(+) CID spectrum of mass selected [**II_3_**]^+^ (*m/z* 589); collision energy voltage 5 V.

CID of the bicyclic Diels–Alder intermediate **[II_3_**]^+^ revealed a fascinating feature (Figure 11). [**II_3_**]^+^ shows two competing fragmentation pathways upon collisional activation. On the one hand, it releases substrate **2** which leads back to the ion [**I_3_**]^+^. This ion subsequently loses CO_2_ as already observed in the CID experiment for [**I_3_**]^+^ (Figure 10). The first pathway thus mimics going back one step in the catalytic cycle. On the other hand, [**II_3_**]^+^ fragments into catalyst **1** by simultaneously cleaving off N_2_ and the product **3**. The second pathway thus reflects going two steps ahead in the catalytic cycle. Such a combination of fragmentation pathways is in perfect accordance with expectations for a reaction intermediate **II_3_** at this position in the catalytic cycle.

**Figure 12:**
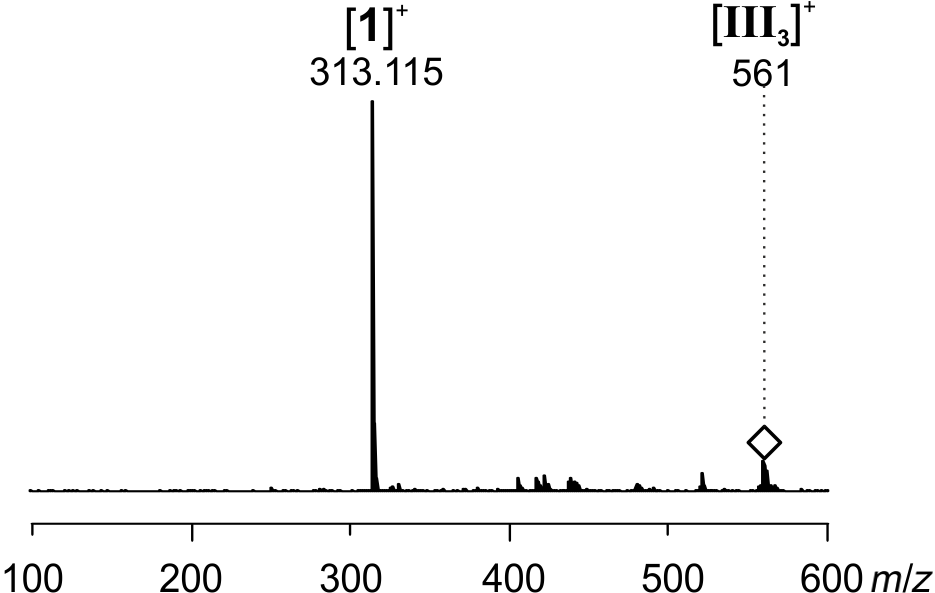
ESI(+) CID spectrum of mass selected [**III_3_**]^+^ (*m/z* 561); collision energy voltage 10 V.

Finally, CID of the mass-selected dihydropyridazine intermediate **III_3_** again leads to catalyst **1** by elimination of product **3** (Figure 12). Thus, also the last step of the catalytic cycle is viable in the gas phase which lends further support to the interpretation of the observed ions as reaction intermediates.

## Conclusion

The L-proline-catalyzed reaction between acetone and 3,6-di(2-pyridyl)-1,2,4,5-tetrazine (**2**) via a Diels–Alder reaction with inverse electron demand was thoroughly studied by ^1^H NMR and ESIMS. Without modification of the substrates, the progress of the reaction could be monitored over time, but only two out of three proposed intermediates of the postulated catalytic cycle [51] could be experimentally detected. The use of a charge-tagged reactant did not lead to better results. However, the charge-tagged proline derivative **1**^+^ designed for an enhanced mass spectrometric detection of low-concentrated intermediates made it possible to detect all relevant species of the reaction including the elusive Diels–Alder intermediate **II_3_**. The direct observation of this ion is the first experimental proof that the reaction is not concerted, but does proceed in a stepwise manner. The three intermediates could be further characterized by collision-induced dissociation in the gas phase. The observed fragmentation pathways mimic the neighboring steps in the catalytic cycle and thus give further support for the intermediate nature of the detected species.

## Experimental

### ESIMS and NMR experiments

All ESIMS experiments were conducted with a micrOTOF-Q mass spectrometer from Bruker Daltonik. Before each measurement, the settings were tuned for high signal intensities. The parameters were adjusted accordingly for each measurement within the following ranges or were constant: end plate offset: −500 V, capillary voltage: −5000 V to −3000 V, nebulizer gas: 1–4 bar, dry gas: 1–3 L/min, dry temperature: 200 °C, collision energy: 1–8 eV, collision RF: 120–155 Vpp, transfer time: 134 µs, pre pulse storage: 8–10 µs, funnel 1 RF: 150–200 Vpp, funnel 2 RF: 150–200 Vpp, hexapole RF: 150 Vpp, ISCID energy: 0 eV, quadrupole ion energy: 1–6 eV. The mass calibration in the MS^2^ spectra was unfortunately slightly shifted. The measured *m*/*z* values are about 35 mDa too high (Figure 10) and about 40 mDa too low (Figure 11 and Figure 12). PEEK tubes with an inner diameter of 0.127 mm and PEEK microreactors (swept volume: 2.2 µL) were used. Airtight glass syringes (250 µL and 5 mL) from Hamilton and syringe pumps (single and double) from Cole Parmer were used.

All kinetic ^1^H NMR experiments were conducted with a Bruker Avance III HD Ascend 700 MHz spectrometer equipped with a 5 mm QCI H-P/C/N cryoprobe with Z-gradient coils. The sample was shimmed before the first measurement. Then, spectra were measured with only one scan in defined intervals without ejecting the sample tube from the instrument.

### Reaction R1 (acetone, untagged substrate, L-proline, rt)

#### Experiment 1 NMR

A solution of L-proline in deuterated dimethyl sulfoxide (1.206 mL, 0.58 mmol/L, 0.0007 mmol, 0.05 equiv) was added to commercially available 3,6-di(2-pyridyl)-1,2,4,5-tetrazine (**2**, 3.3 mg, 0.014 mmol, 1.0 equiv). Additional 1.764 mL of deuterated dimethyl sulfoxide were added. The concentration was chosen sufficiently low to ensure that all components were fully dissolved at room temperature. A solution of acetone in deuterated dimethyl sulfoxide solution was added last (41 µL, 1.4 mmol/mL, 0.057 mmol, 4 equiv) to start the reaction. The first spectrum was measured 6:14 minutes after the start of the reaction. Then spectra were measured in intervals of 5 minutes at room temperature. The plotted signal intensities were normalized to the dimethyl sulfoxide solvent peak.

#### Experiment 2 ESIMS

Commercially available 3,6-di(2-pyridyl)-1,2,4,5-tetrazine (**2**, 50 mg, 0.20 mmol, 1.0 equiv) and L*-*proline (1.2 mg, 0.01 mmol, 0.05 equiv) were mixed in 10 mL dimethyl sulfoxide. Acetone (63 µL, 0.8 mmol, 4 equiv) was added last. In regular intervals, samples of 1 µL were taken directly from the reaction mixture and immediately diluted in 0.5 mL acetonitrile. The diluted samples were fed into the mass spectrometer in a timely manner with a flow rate of 300 µL/h.

#### Experiment 3 ESIMS (enamine preformation)

L*-*proline (1.2 mg, 0.01 mmol, 1.0 equiv) and acetone (72 µL, 0.97 mmol, 95 equiv) were mixed in 5 mL dimethyl sulfoxide and stirred at room temperature for 10 min. The preformation of enamine **I_1_** was validated with ESIMS. Afterwards, commercially available 3,6-di(2-pyridyl)-1,2,4,5-tetrazine (**2**, 2.4 mg, 0.01 mmol, 1.0 equiv) was added. In regular intervals, samples of 20 µL were taken directly from the reaction mixture and immediately diluted in 0.5 mL acetonitrile. The diluted samples were fed into the mass spectrometer in a timely manner with a flow rate of 200 µL/h.

### Reaction R2 (acetone, charge-tagged substrate, L-proline, rt) ESIMS

A 0.4 mmol/L stock solution of **4**∙Br in dimethyl sulfoxide was prepared (stock solution ss_1_), as well as a 0.001 mmol/L stock solution of L*-*proline in dimethyl sulfoxide/acetone 1:1 (stock solution ss_2_).

#### Continuous flow setup

A continuous flow setup [4,17–18] was used for the experiment. A schematic depiction of the setup can be found in Scheme S2, Supporting Information File 1. An airtight syringe s_1_ was loaded with stock solution ss_1_ and syringe s_2_ was loaded with stock solution ss_2_. By using a double syringe pump, the contents of s_1_ and s_2_ were pumped via PEEK tubes with a flow rate f_A_ = 75 µL/h into microreactor m_A_, where they were mixed. From microreactor m_A_ the combined solutions flowed towards microreactor m_B_ where they were diluted with DMSO, which was injected with flow rate f_B_ = 150 µL/h. The outlet of microreactor m_B_ was directly fed into the spectrometer with an effective flow rate of f_B_ + 2 × f_A_ = 300 µL/h. The theoretical reaction time was calculated. Further details on the calculation can be found in Supporting Information File 1.

#### Syringe setup

1 mL (0.0004 mmol, 1 equiv of **4**∙Br) of stock solution ss_1_ was mixed with 2 mL dimethyl sulfoxide and 1 mL of stock solution ss_2_, which contained 0.001 mmol, 4 equiv of L-proline and 0.5 mL of acetone.

A 5 mL syringe was charged with the combined solution and fed it into the ESI source of the mass spectrometer over a period of 4 h with a flow rate of 400 µL/h, while spectra were taken continuously.

### Reaction R3 (acetone, untagged substrate, charge-tagged catalyst, rt to 60 °C) ESIMS

Commercially available 3,6-di(2-pyridyl)-1,2,4,5-tetrazine (**2**, 19 mg, 0.08 mmol, 1 equiv) and **1**∙Cl (6 mg, 0.02 mmol, 0.2 equiv) were mixed in 1.13 mL dimethyl sulfoxide and 0.2 mL methanol. Acetone (600 µL, 3.87 mmol, 50 equiv) was added last. During the experiment the temperature was slowly raised from room temperature up to 60 °C (see temperature curve in Figure 7 and Figure S3, Supporting Information File 1). In regular intervals, samples of 2 µL were taken directly from the reaction mixture and immediately diluted with 0.5 mL of a (1:1) mixture of methanol and acetonitrile. The diluted samples were fed into the ESI source of the mass spectrometer in a timely manner with a flow rate of 300 µL/h.

A CID experiment of **1** has been performed (Figure S1, Supporting Information File 1).

### Synthesis

All ratios given are volume ratios unless stated otherwise. Commercially available chemicals were used without prior purification. Solvents (cyclohexane, dichloromethane, ethyl acetate) were dried with standardized methods. Inert gas atmosphere reactions were performed under argon using standard Schlenk techniques and oven-dried glassware prior to use. Thin-layer chromatography was performed with TLC plates form Merck (aluminum sheets silica gel 60 F_254_) and detection was performed by fluorescent light λ = 245 nm and λ = 366 nm. Purification of products by column chromatography was done on silica gel 60, 40–63 μm from Merck. For ^1^H and ^13^C NMR analysis a Bruker Avance I 400 MHz instrument was used with 400 MHz for ^1^H spectra, 162 MHz for ^31^P spectra and 101 MHz for ^13^C spectra or a Bruker Avance I 500 MHz instrument was used with 500 MHz for ^1^H spectra and with 126 MHz for ^13^C spectra. The allocation of NMR signals was accomplished with H,H-COSY, HMBC or HSQC spectra. Deuterated solvents chloroform-*d*_1_ and DMSO-*d*_6_ were obtained from Deutero GmbH and the remaining non-deuterated solvent signals were used as internal standards as references for the ^1^H shifts and ^13^C shifts which are all reported on the δ [ppm] scale. UV–vis spectra were measured on a Lambda 18 instrument from Perkin Elmer and fluorescence spectra were measured on a LS50B instrument from Perkin Elmer. All EI spectra were measured on a MAT 95 XL instrument from Thermo Finnigan and ESI spectra of synthesized compounds were measured with either the micrOTOF-Q instrument from Bruker Daltonik GmbH or with an Orbitrap XL instrument from Thermo Fisher Scientific.

#### Charge-tagged catalyst (**1**∙Cl)

The charge-tagged catalyst **1**∙Cl was synthesized according to the protocol of Willms et al. [18].

#### 1-Benzoyl-2-*p*-toluoylhydrazide (**6**)

Under argon atmosphere benzhydrazide (0.09 g, 0.64 mmol, 1.0 equiv) was dissolved in dichloromethane (6.5 mL) and within 40 min *p*-toluoyl chloride (0.11 g, 0.71 mmol, 1.1 equiv) in dichloromethane (1.3 mL) was added dropwise under constant stirring. A colorless solid precipitated. The suspension was stirred for 1 h at room temperature. The solid was filtered off and washed with dichloromethane (10 mL) and dried in vacuo. 0.12 g of raw product were obtained and purified via column chromatography (ethyl acetate/cyclohexane 4:1, *R*_f_ = 0.91). 0.10 g of colorless solid were obtained. The protocol has been adapted from Wang et al. [55]. Yield: 0.10 g (0.39 mmol, 83%), ^1^H NMR (400 MHz, DMSO-*d*_6_, 298 K) δ [ppm] 10.46 (s, 2H, H-7, H-8), 7.96–7.93 (m, 2H, H-11), 7.85 (pd, 2H, H-4), 7.62–7.57 (m, 1H, H-13), 7.55–7.50 (m, 2H, H-12), 7.33 (pd, 2H, H-3), 2.38 (s, 3H, H-1); ^13^C{^1^H} NMR (101 MHz, DMSO-*d*_6_, 298 K) δ [ppm] 165.9 (C-9), 165.8 (C-6), 141.9 (C-2), 132.6 (C-13), 131.8 (C-10), 129.8 (C-5), 129.0 (C-3), 128.5 (C-12), 127.51 (C-4), 127.47 (C-11), 21.1 (C-1). The numbering of the atoms in the molecule can be found in Supporting Information File 1. The allocation of signals has been done with HMBC and HSQC spectra. HRESIMS(+) *m*/*z*: [M + Na]^+^ calcd for C_15_H_14_N_2_O_2_Na, 277.0947; found, 277.0981.

#### *N*-(Chloro(phenyl)methylene)-4-methylbenzohydrazonoyl chloride (**7**)

Under argon atmosphere **6** (1.00 g, 7.34 mmol, 1.0 equiv) was dissolved in toluene (50 mL) and phosphorous pentachloride (8.05 g, 36.72 mmol, 5.0 equiv) was added. The mixture was stirred at reflux conditions for 3 h. The solvent was distilled of in vacuo at 40 °C. The raw product (0.65 g) was purified via column chromatography (cyclohexane/dichloromethane, 100:1, *R*_f_ = 0.23). A yellow solid was obtained (0.48 g). The protocol has been adapted from Wang et al. [55]. Yield: 0.48 g (1.65 mmol, 48%), ^1^H NMR (400 MHz, CDCl_3_, 298 K) δ [ppm] 8.17–8.14 (m, 2H, H-9), 8.04 (pd, 2H, H-4), 7.56–7.52 (m, 1H, H-11), 7.51–7.46 (m, 2H, H-10), 7.29 (pd, 2H, H-3), 2.44 (s, 3H, H-1); ^13^C{^1^H} NMR (101 MHz, CDCl_3_, 298 K) δ [ppm] 144.6 (C-7), 144.3 (C-6), 142.6 (C-2), 133.9 (C-8), 131.9 (C-11), 131.1 (C-5), 129.4 (C-3), 128.69 (C-4, C-9), 128.65 (C-10), 21.7 (C-1). The numbering of the atoms in the molecule can be found in Supporting Information File 1. The allocation of signals has been done with HMBC and HSQC spectra; EIMS (70 eV) *m/z* (%): 290.0 (69) [M]^+•^, 255.0 (69) [M − Cl]^+^, 152.0 (100) [M − C_8_H_7_Cl]^+•^, 138.0 (47), 117.0 (38), 103.0 (39), 91.0 (42) [C_7_H_7_]^+^, 77.0 (39) ) [C_6_H_5_]^+^.

#### 3-(4-Methylphenyl)-6-phenyl-1,2,4,5-tetrazine (**8**)

**7** (434 mg, 1.66 mmol, 1 equiv) was dissolved in 11 mL acetonitrile and hydrazine (98 µL, 1.66 mmol, 1 equiv) was added. The mixture was refluxed for 1 h behind a blast shield. Then potassium carbonate (412 mg, 3.31 mmol, 2 equiv) was added and the mixture was refluxed for another 24 h. Hydrazine (587 µL, 9.93 mmol, 6 equiv) was added again and the mixture was refluxed for 1 h. When the mixture had cooled to room temperature 10 mL of dichloromethane were added. The organic layer was washed with brine and dried over magnesium sulfate. The solvents were evaporated and the remaining solid was dissolved in 4.4 mL acetic acid at 0 °C. The mixture was stirred while a solution of sodium nitrite (839 mg, 12.17 mmol, 7.4 equiv) in 1 mL of deionized water was added dropwise. The mixture was stirred for another 3 h, after the solution of sodium nitrite had been added. 55 mL dichloromethane were added and the organic layer was washed twice with saturated sodium hydrogen carbonate solution and dried over magnesium sulfate. The solvents were evaporated and 340 mg of a pink raw product were purified with column chromatography (cyclohexane/dichloromethane 7:3, *R*_f_ = 0.41). 193 mg of a pink solid were obtained. Yield: 193 mg (0.78 mmol, 52%), ^1^H NMR (400 MHz, CDCl_3_, 298 K) δ [ppm] 8.66–8.62 (m, 2H, H-9), 8.54 (pd, 2H, H-4), 7.66–7.58 (m, 3H, H-10 and H-11), 7.42 (pd, 2H, H-3), 2.48 (s, 3H, H-1); ^13^C{^1^H} NMR (101 MHz, CDCl_3_, 298 K) δ [ppm] 164.1 (C-6), 163.9 (C-7), 143.6 (C-2), 132.7 (C-11), 132.0 (C-8), 130.2 (C-3), 129.4 (C-10), 129.2 (C-5), 128.1 (C-4), 128.0 (C-9), 21.9 (C-1). The numbering of the atoms in the molecule can be found in Supporting Information File 1. The allocation of NMR signals was accomplished with H,H-COSY, HMBC and HSQC spectra; HREIMS: [M]^+•^ calcd for C_15_H_12_N_4_, 248.1062; found, 248.1059.

#### 3-(4-Bromomethylphenyl)-6-phenyl-1,2,4,5-tetrazine (**9**)

**9** was prepared in a slight modification of the protocol used by de Almeida et al. [57]. **8** (76 mg, 0.31 mmol, 1 equiv) and TBCA (tribromoisocyanuric acid, 336 mg, 0.92 mmol, 3 equiv) were refluxed in 3 mL ethyl acetate for six hours. The precipitated cyanuric acid was filtered off over Celite^®^. The solvent was evaporated and 102 mg of a pink raw product was obtained. The raw product was purified via column chromatography (cyclohexane/ethyl acetate, 10:0.25, *R*_f_ = 0.1) and 45 mg of a pink product were obtained. Yield: 45 mg (0.14 mmol, 45%), ^1^H NMR (500 MHz, CDCl_3_, 298 K) δ [ppm] 8.67–8.63 (m, 4H, H-4, H-9), 7.67–7.60 (m, 5H, H-3, H-11, H-10), 4.58 (s, 2H, H-1); ^13^C{^1^H} NMR 126 MHz, CDCl_3_, 298 K) δ [ppm] 164.1 and 163.7 (C-6, C-7)*, 142.6 (C-2), 132.9 (C-11), 131.9 and 131.9 (C-5, C-8)*, 130.1 (C-3), 129.5 (C-10), 128.5 and 128.2 (C-4, C-9)*, 32.5 (C-1). The numbering of the atoms in the molecule can be found in Supporting Information File 1. *The two signals can only be allocated to either two carbon atoms. The allocation of NMR signals was accomplished with H,H-COSY, HMBC and HSQC spectra. UV–vis: 307.5 nm global maximum, 224.5 nm local maximum, 549 nm local maximum. Fluorescence (excitation 307 nm): 358.5 nm global maximum, 610.5 local maximum. HREIMS: [M]^+•^ calcd for C_15_H_11_BrN_4_, 326.0167; found, 326.0165; EIMS *m/z* (%): 326.0 (3%) [M]^+•^, 247.1 (4%) [M − Br]^+^, 116.0 (100%) [M − C_7_H_5_BrN_3_]^+^, 103.0 (36%), 76.0 (7%).

#### TBCA preparation

The synthesis of tribromoisocyanuric acid was conducted according to the procedure of de Almeida et al. [57]. A solution of OXONE^®^ (14.29 g, 46.49 mmol, 3 equiv, ingredients see below) in 186 mL deionized water was added dropwise to a 0 °C cold stirred solution of cyanuric acid (2.00 g, 15.50 mmol, 1 equiv), sodium hydroxide (1.86 g, 46.49 mmol, 3 equiv), sodium carbonate (2.46 g, 23.24 mmol, 1.5 equiv) and potassium bromide (5.53 g, 46.49 mmol, 3 equiv) in 223 mL deionized water within 2 h. The solution was stirred at room temperature for 24 h. The precipitated white solid was filtered off and washed with cold deionized water. TBCA was directly used for the synthesis of **9** without further treatment.

#### Triphenyl[4-(6-phenyl-1,2,4,5-tetrazin-3-yl)benzyl]phosphonium bromide (**4**∙Br)

**4**∙Br was prepared in a slight modification of the protocol published by Vikse et al. [53]. Under argon atmosphere **9** (40 mg, 0.12 mmol, 1 equiv) and triphenylphosphane (67 mg, 0.18 mmol, 1.5 equiv) were mixed in 0.47 mL toluene and stirred at 50 °C for 24 h. Dichloromethane (15 mL) was added and the organic layer was washed six times with a 2:1 mixture of deionized water/methanol (6 × 40 mL). The aqueous layer was evaporated to yield 17 mg of a light pink solid. The ^1^H NMR, ^31^P NMR and ESIMS spectra show an impurity of triphenylphosphine oxide in the product, which does not interfere with the ESIMS experiment in which **4**∙Br was used as the charge-tagged substrate. Yield: 17 mg (0.03 mmol, ≈27% including impurity), ^1^H NMR (400 MHz, CDCl_3_, 298 K) δ [ppm] 8.54–8.52 (m, 2H, H-4), 8.26–8.24 (m, 2H, H-9), 7.88–7.84 (m, 6H, H-14), 7.78–7.75 (m, 3H, H-16), 7.69–7.58 (m, 9H, 6H of the 9H correlate to H-15, rest correlates to impurity of POPh_3_), 7.55–7.52 (m, 2H, H-3), 7.48–7.45 (m, 1H, H-11), 7.40–7.37 (m, 2H, H-10), 5.85 (d, 2H, H-1); ^13^C{^1^H} NMR (126 MHz, CDCl_3_, 298 K) δ [ppm] 164.0 (C-6), 163.6 (C-7), 135.1 or 135.1 (C 13), 134.8 (C-14), 134.7 (C-16), 132.8 or 132.8 or 132.8 (C-5 and C-8), 130.4 or 130.3 (C-15), 129.4 (C-3), 128.7 (C-10), 128.6 (C-11), 128.1 (C-4), 128.1 (C-9), 128.1, 118.3 (C-2), 117.6 (C-2), 31.0 or 30.6 (C-1). Signals not allocated to **4**∙Br: 132.9, 132.7, 132.3, 132.2, 132.1, 131.7, 131.7, 131.6; ^31^P{^1^H} NMR (162 MHz, CDCl_3_, 298 K) δ [ppm] 29.22 (triphenylphosphine oxide), 23.72 (P-12). The numbering of the atoms in the molecule can be found in Supporting Information File 1. The allocation of NMR signals was accomplished with H,H-COSY, HMBC and HSQC spectra. UV–vis: local maximum ≈240 nm, local maximum ≈300 nm, local maximum 548.5 nm. Fluorescence (excitation 302 nm): 368.0 nm global maximum. HRESIMS: [M]^+^ calcd for C_33_H_26_N_4_P^+^, 509.1890; found, 509.1884. ESI-CID (Figure S4, Supporting Information File 1).

## Supporting Information

File 1 Additional material.
