# Supplementary material for: Mechanistic studies of an L-proline-catalyzed pyridazine formation involving a Diels–Alder reaction with inverse electron demand
Source: Beilstein J Org Chem. 2019 Jan 3;15:30–43. doi: 10.3762/bjoc.15.3 (PMC6334817; doi:10.3762/bjoc.15.3)
Supplement: File 1 — Additional material. [file Beilstein_J_Org_Chem-15-30-s001.pdf]

BEILSTEIN JOURNAL OF ORGANIC CHEMISTRY

Supporting Information

for

Mechanistic studies of an L-proline-catalyzed pyridazine formation involving a Diels–Alder reaction with inverse electron demand

Anne Schnell, J. Alexander Willms, S. Nozinovic and Marianne Engeser

Beilstein J. Org. Chem. **2019,** *15,* 30–43. doi:10.3762/bjoc.15.3

Additional material

License and Terms: This is a supporting information file under the terms of the Creative Commons Attribution License (http://creativecommons.org/licenses/by/4.0). Please note that the reuse, redistribution and reproduction in particular requires that the authors and source are credited.
The license is subject to the *Beilstein Journal of Organic Chemistry* terms and conditions: (https://www.beilstein-journals.org/bjoc)

Table of Contents:

Reaction R1:

^{1}H NMR spectra of NMR experiment with low concentration page S3,S4

^{1}H NMR spectrum of acetone and L-proline page S5

Experimental description of NMR experiment at higher concentration page S6

Temporal progress of NMR experiment at higher concentration page S6

Experimental description of ESIMS experiment at low concentration page S7

Temporal progress of ESIMS experiment at low concentration page S7

Reaction R2:

Scheme S1: Two possible regioisomers of the product **5**·Br page S8

Scheme S2: Schematic depiction of continuous flow setup page S9

Calculations of reaction time of continuous flow setup page S9

Reaction R3:

CID spectrum of charge tagged catalyst $\mathbf{1}^{+}$ page S10

CID spectrum of adduct $[\mathbf{1}+\mathbf{2}]^{+}$ at *m*/*z* 549 page S11

Synthesis

Synthetic products with numbering used for NMR signal allocation page S11-S13

Accurate mass determination with Orbitrap XL page S13-S18

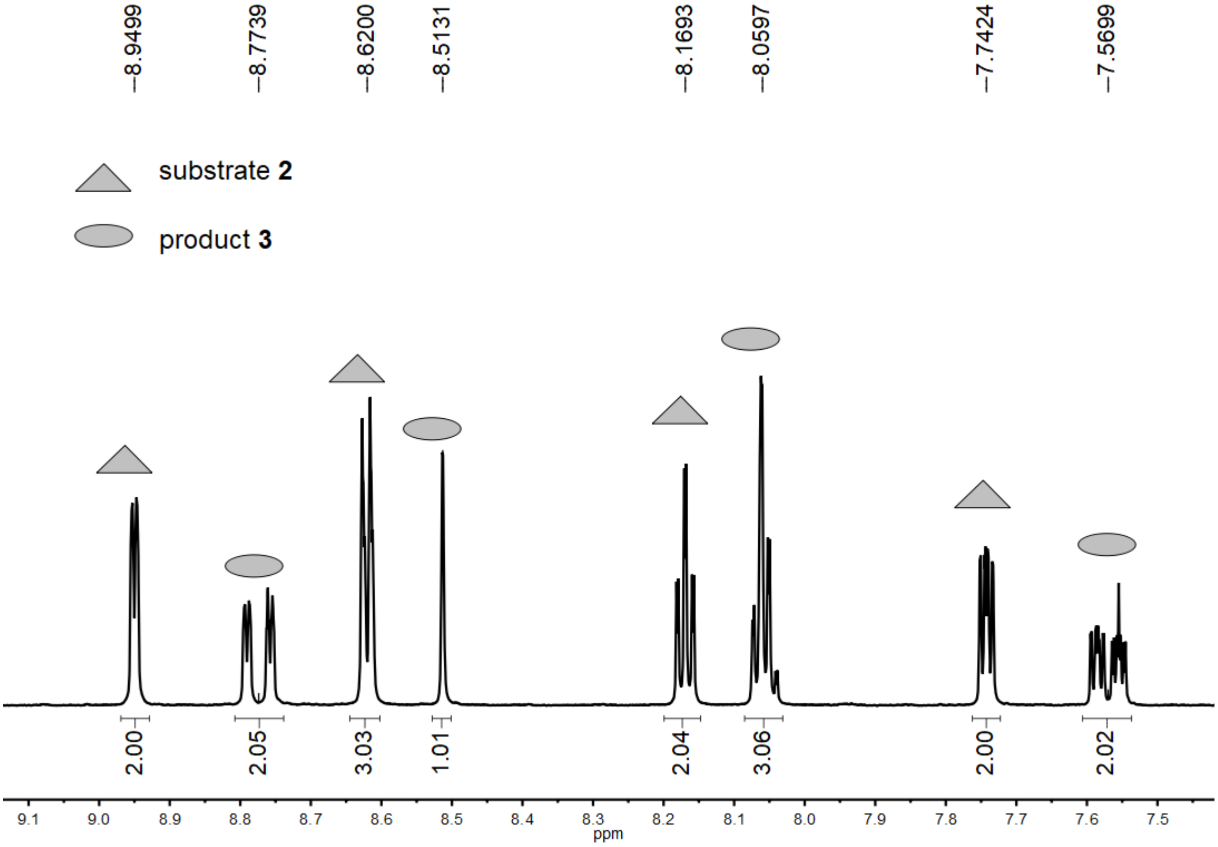

Figure S1: ^{1}H NMR spectrum (aromatic region) during the NMR experiment of reaction R1 with low concentration (concentration of tetrazine 0.005 mmol/mL) after 3 h and 50% conversion.

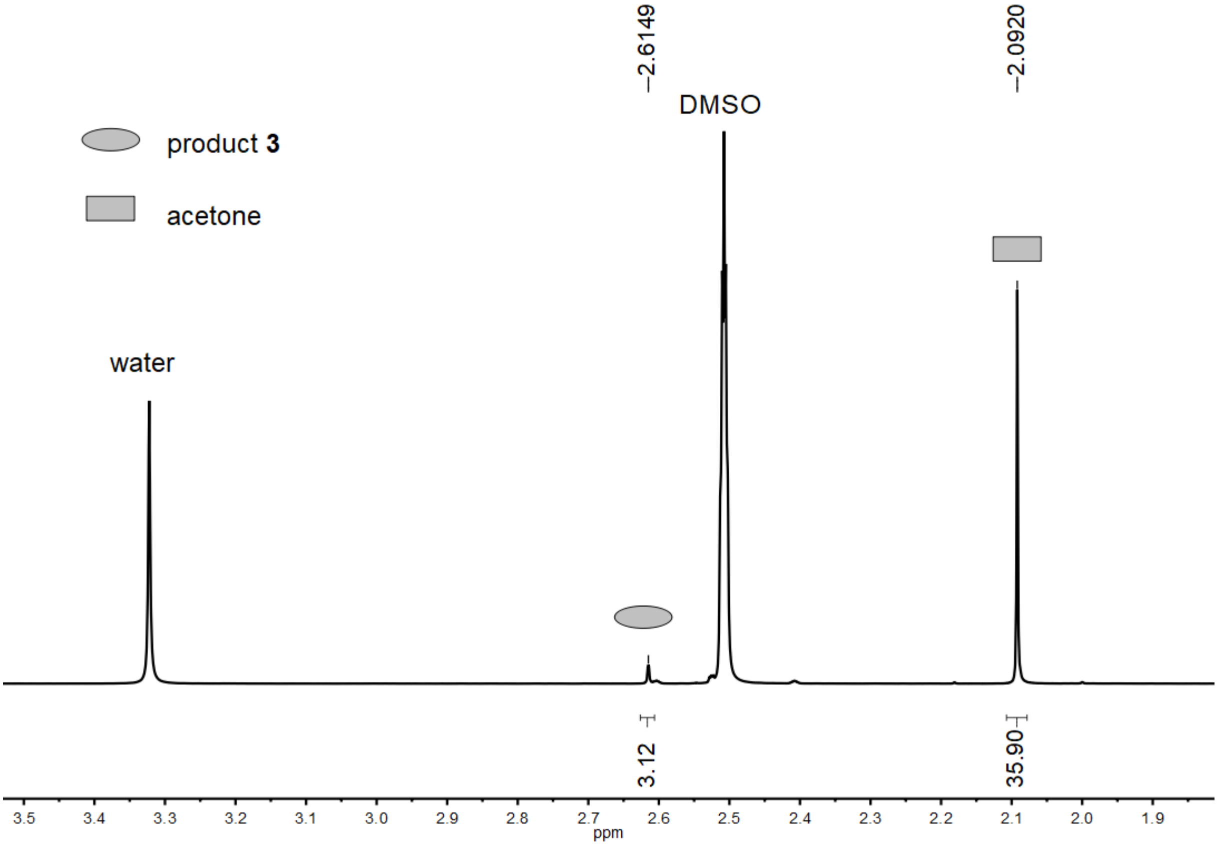

Figure S2: ^{1}H NMR spectrum (aliphatic region) during the NMR experiment of reaction R1 with low concentration (concentration of tetrazine 0.005 mmol/mL) after 3 h and 50% conversion.

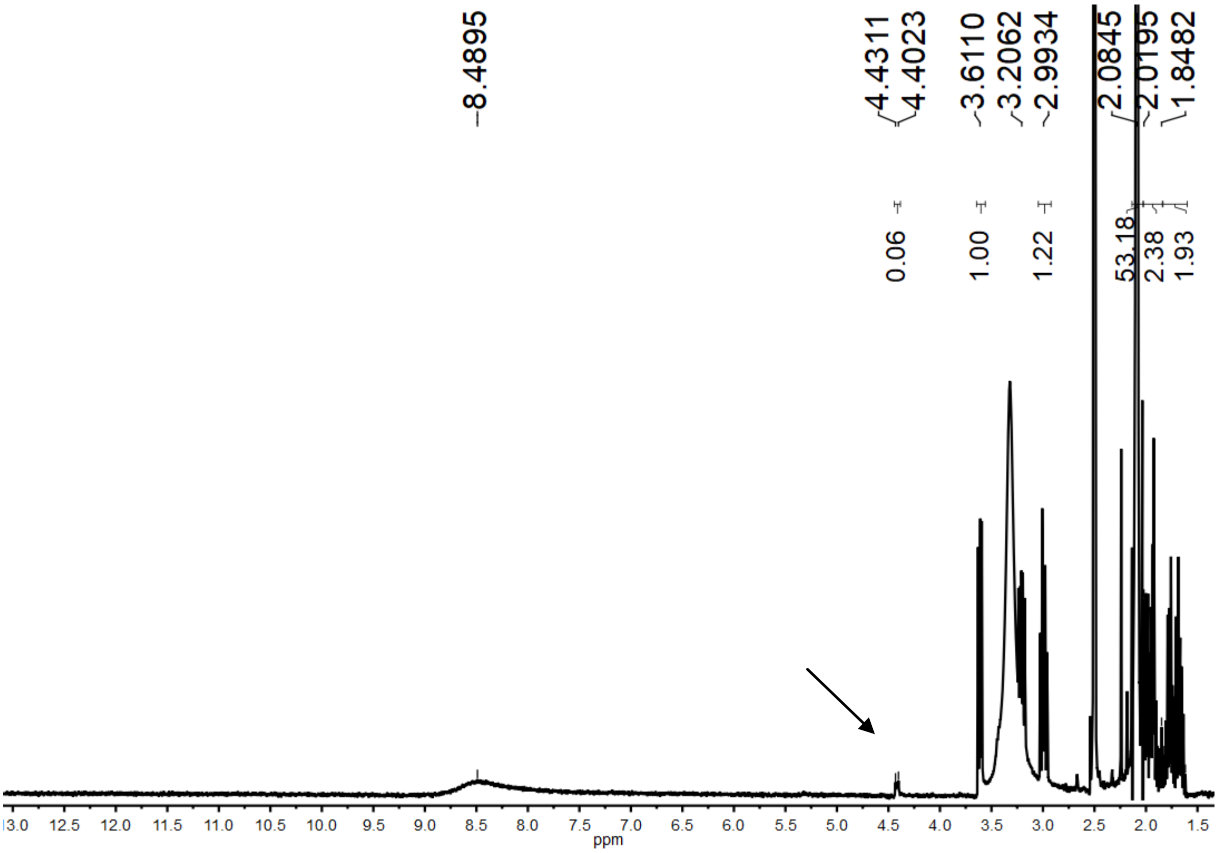

Figure S3: ^{1}H NMR spectrum of the reaction mixture in absence of tetrazine, i.e., a mixture of acetone (1 equiv) and L-proline (0.01 equiv) in deuterated dimethyl sulfoxide. The arrow points at the signal at 4.4 ppm which is the significant signal for the oxazolidinone as published by List [1] and Gschwind [2]. The spectrum was measured with a Bruker Avance I 400 MHz NMR spectrometer.

NMR study of reaction R1 at higher concentrations:

Commercially available 3,6-di-2-pyridyl-1,2,4,5-tetrazine **2** (28.3 mg, 0.12 mmol, 1.0 equiv) and L-proline (0.69 mg, 0.006 mmol, 0.05 equiv) were mixed in 3.6 mL of deuterated dimethyl sulfoxide. 0.5 mL of this mixture were transferred to the NMR sample tube. To start the reaction, a solution of acetone in deuterated dimethyl sulfoxide was added (50 µL, 9.6 mmol/ml, 0.48 mmol, 4 equiv). The first spectrum was measured 3 minutes after the start of the reaction. Subsequent spectra were measured in regular intervals of 1 minute at rt.

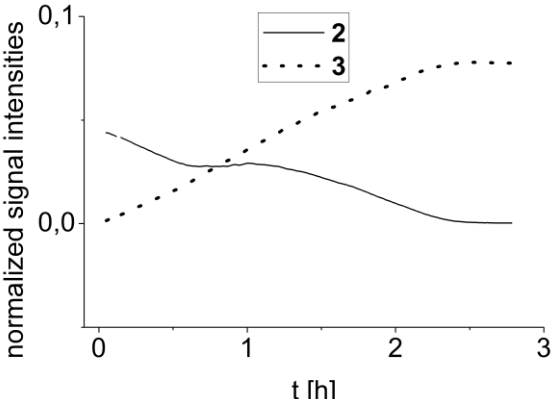

Figure S4: NMR study of reaction R1 at higher concentrations (concentration of tetrazine 0.033 mmol/mL). Product **3** is completely soluble at the given concentration, whereas substrate **2** is only partially dissolved. While substrate **2** transforms into the product **3**,' more of substrate **2** gets dissolved until full conversion is reached.

ESIMS study of reaction R1 at lower concentrations:

A solution of L-proline in dimethyl sulfoxide (1.353 mL, 0.76 mmol/L, 0.001 mmol, 0.05 equiv) is added to commercially available 3,6-di-2-pyridyl-1,2,4,5-tetrazine **2** (4.86 mg, 0.02 mmol, 1.0 equiv). Additional 3.02 mL of dimethyl sulfoxide were added. A solution of acetone in dimethyl sulfoxide was added last (37 µL, 1.1 mmol/mL, 0.08 mmol, 4 equiv). The first spectrum was measured 2:20 minutes after the start of the reaction, subsequent measurements were recorded in intervals of 7.5 min.

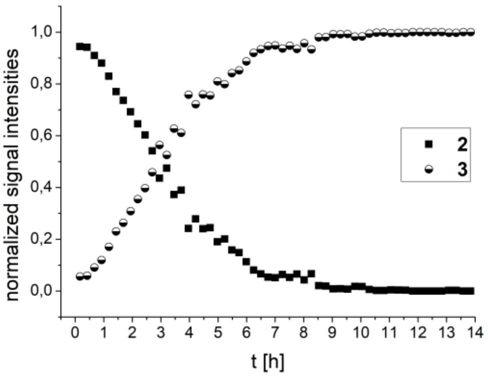

Figure S5: Temporal progress of reaction R1 at low concentrations (0.005 mmol/mL of tetrazine) in ESIMS experiment.

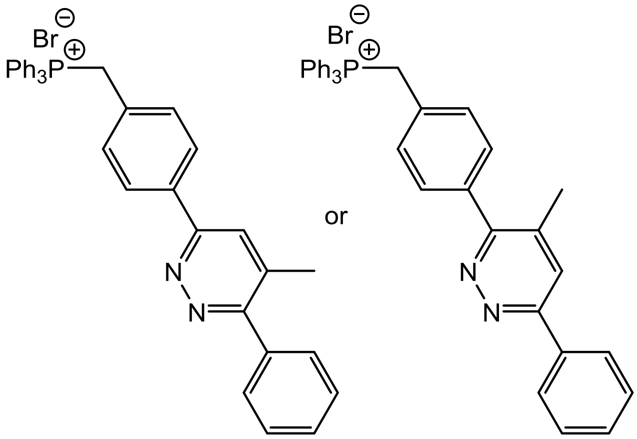

5·Br

Scheme S1: Two possible regioisomers of **5**·Br in reaction R2.

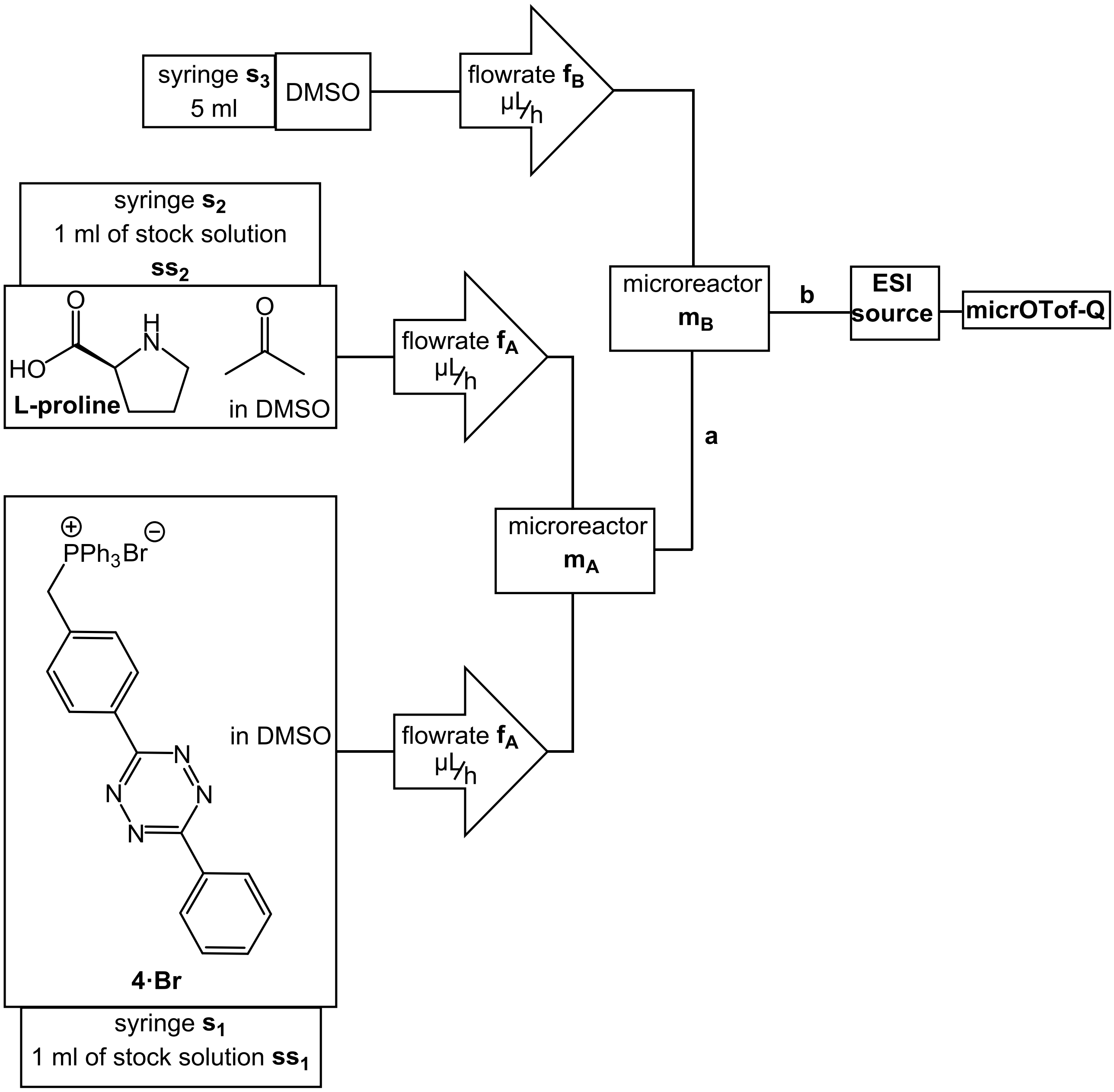

Scheme S2: A schematic depiction of the continuous flow setup for reaction R2.

The theoretical reaction time for the continuous flow setup was calculated by considering the experimental flow rates f_A (150 µL/h) and f_B (300 µL/h). The flowrate in tube a is assumed to be 2^*f_A = 300 µL/h = f_B. Further, the swept volume (V_{swept} =2.2 µL) in the microreactors, the dimensions of the connecting PEEK tubes (length: a = 357 mm, b = 110 mm; diameter d = 0.127 mm) and the volume of the ESI needle (V_{needle} = 0.0069115 µL) is needed. Furthermore, the assumption was made that because the

microreactor m_b dilutes the reaction mixture by half, the reaction rate decreases according to the behavior of a bimolecular reaction, namely resulting in a fourth of the original reaction rate. The volumina V_a and V_b are defined as

$$V_a = a * \left(\frac{d}{2}\right)^2 * \pi + V_{swept}$$

$$V_b = b * \left(\frac{d}{2}\right)^2 * \pi + V_{swept} + V_{needle}$$

and in combination with the flowrates the reaction time can be calculated.

$$t = \frac{V_a}{2 * f_a} + \frac{V_b}{2 * f_b} * 0.25 = \frac{a * \left(\frac{d}{2}\right)^2 * \pi + V_{swept}}{2 * f_a} + \frac{b * \left(\frac{d}{2}\right)^2 * \pi + V_{swept} + V_{needle}}{2 * f_b} * 0{,}25$$

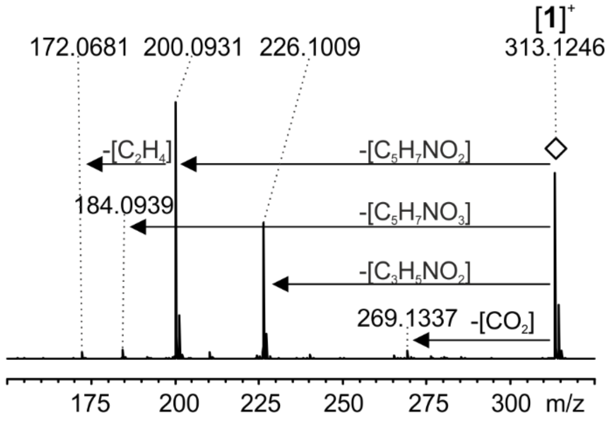

Figure S6: ESI(+) CID spectrum of mass selected [**1**]$^+$ (m/z 313); collisional energy voltage 15 V.

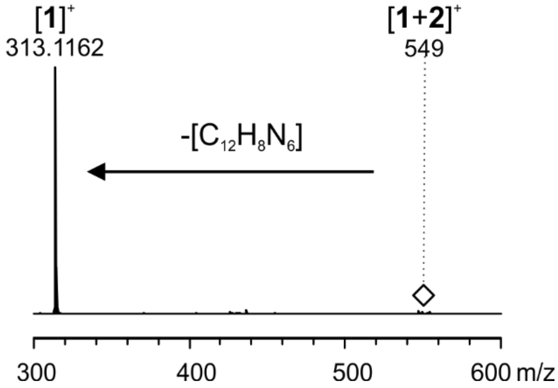

Figure S7: ESI(+) CID spectrum of the mass selected non-covalent adduct [**1**+**2**]$^+$ (*m*/*z* 549); collisional energy voltage 1 V.

Synthesis

Depiction of synthetic products including the numbering used for the NMR signal allocation.

1-Benzoyl-2-*p*-toluoylhydrazide (6)

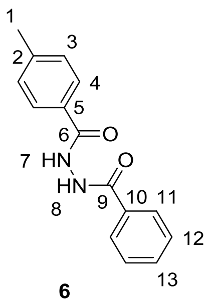

6

N-(Chlor(phenyl)methylene)-4-methylbenzohydrazonoyl chloride (7)

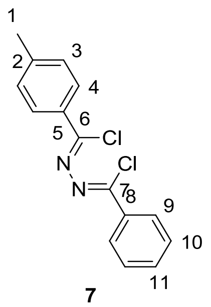

7

3-(4-Methylphenyl)-6-phenyl-1,2,4,5-tetrazine (8)

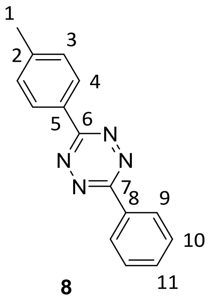

8

3-(4-Bromomethylphenyl)-6-phenyl-1,2,4,5-tetrazine (9)

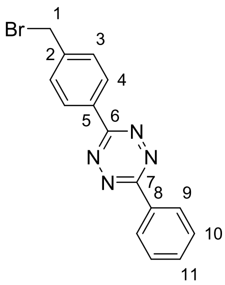

9

3-(4-Triphenylphosphoniummethylphenyl)-6-phenyl-1,2,4,5-tetrazine (4·Br)

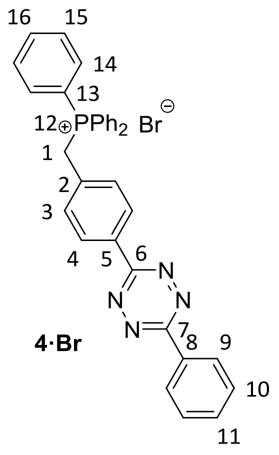

Accurate mass determination with Orbitrap XL

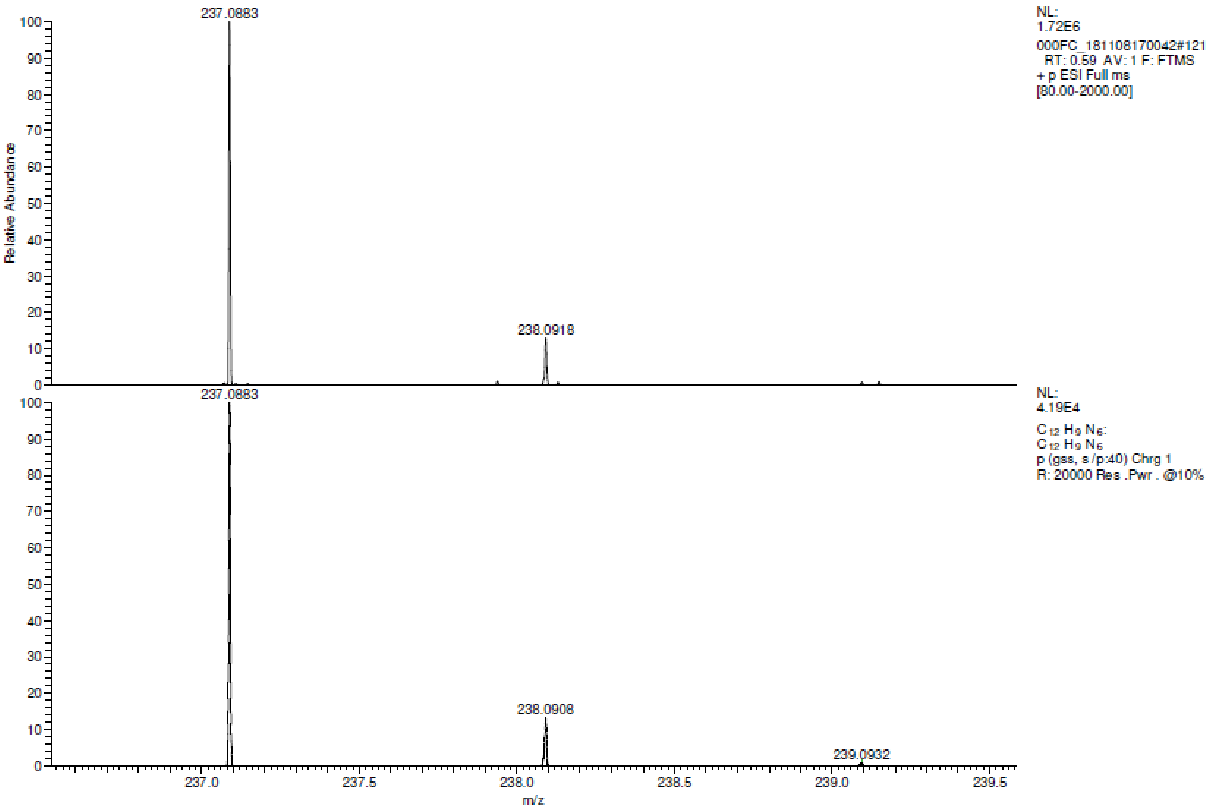

Figure S8: Accurate mass determination with Orbitrap XL of substrate **2** ([**2**+H]$^+$).

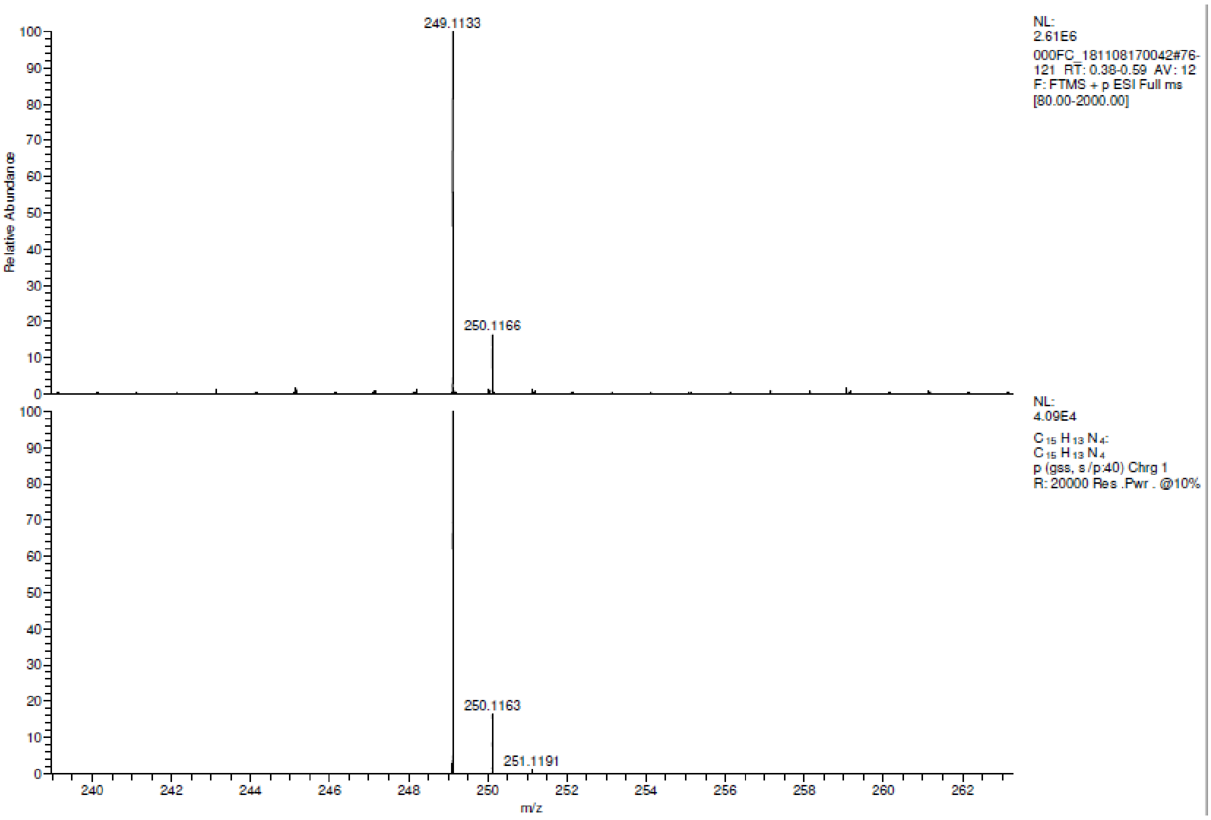

Figure S9: Accurate mass determination with Orbitrap XL of product **3** ([**3**+H]$^+$).

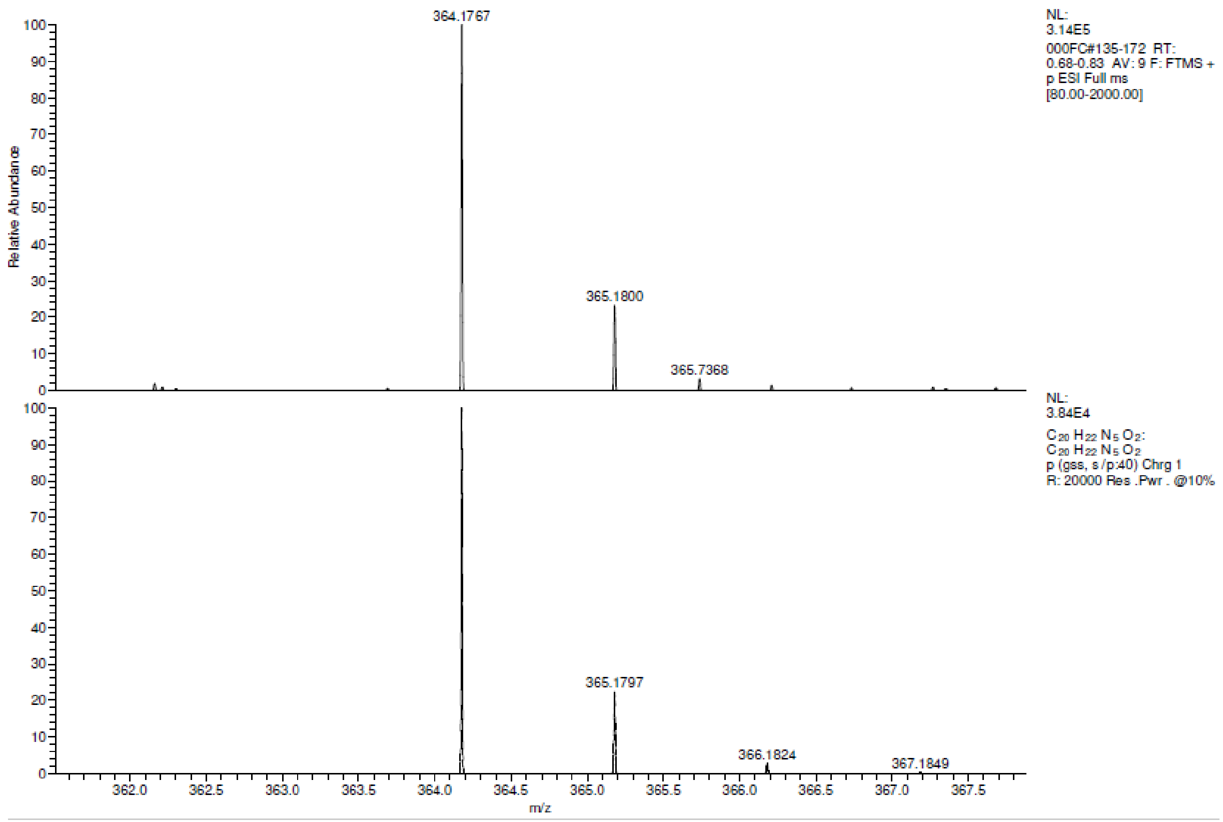

Figure S10 Accurate mass determination with Orbitrap XL of intermediate **III_1** ([**III_1**+H]$^+$).

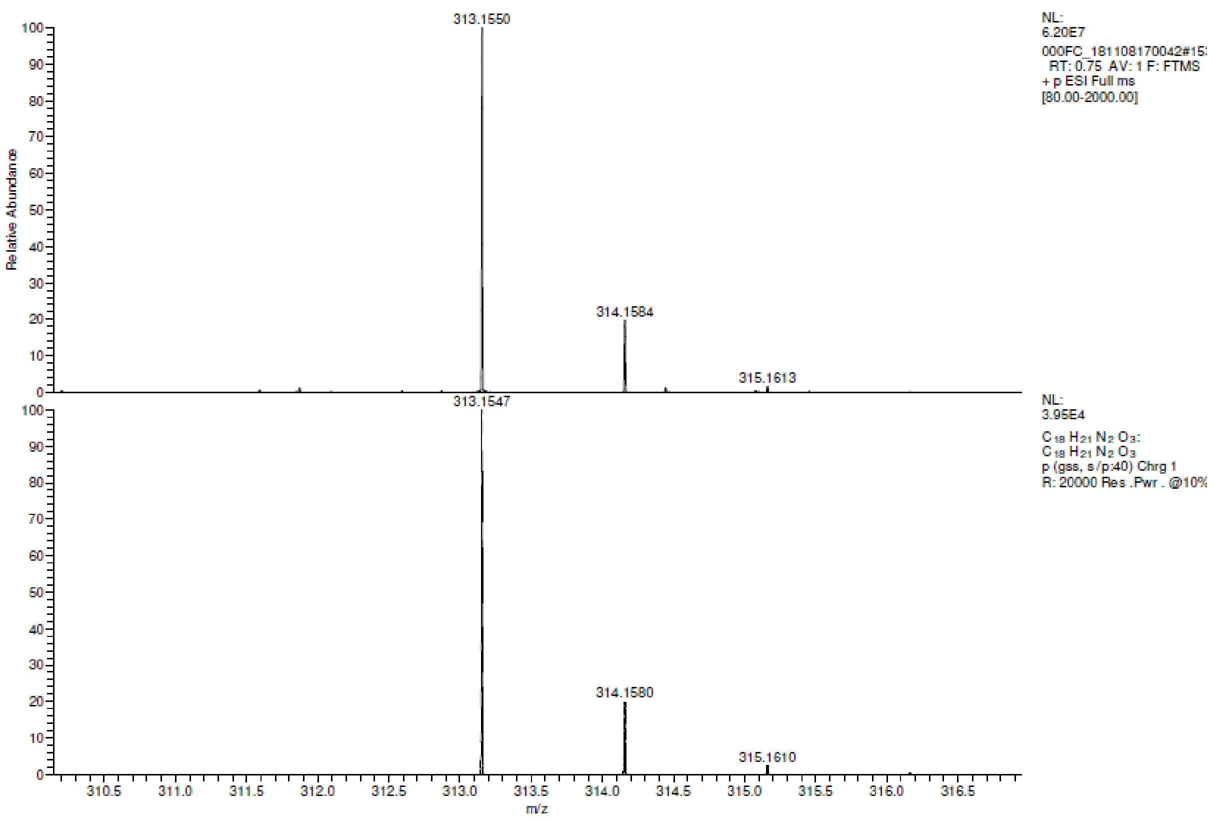

Figure S11: Accurate mass determination with Orbitrap XL of charge tagged catalyst **1** ([**1**]$^+$).

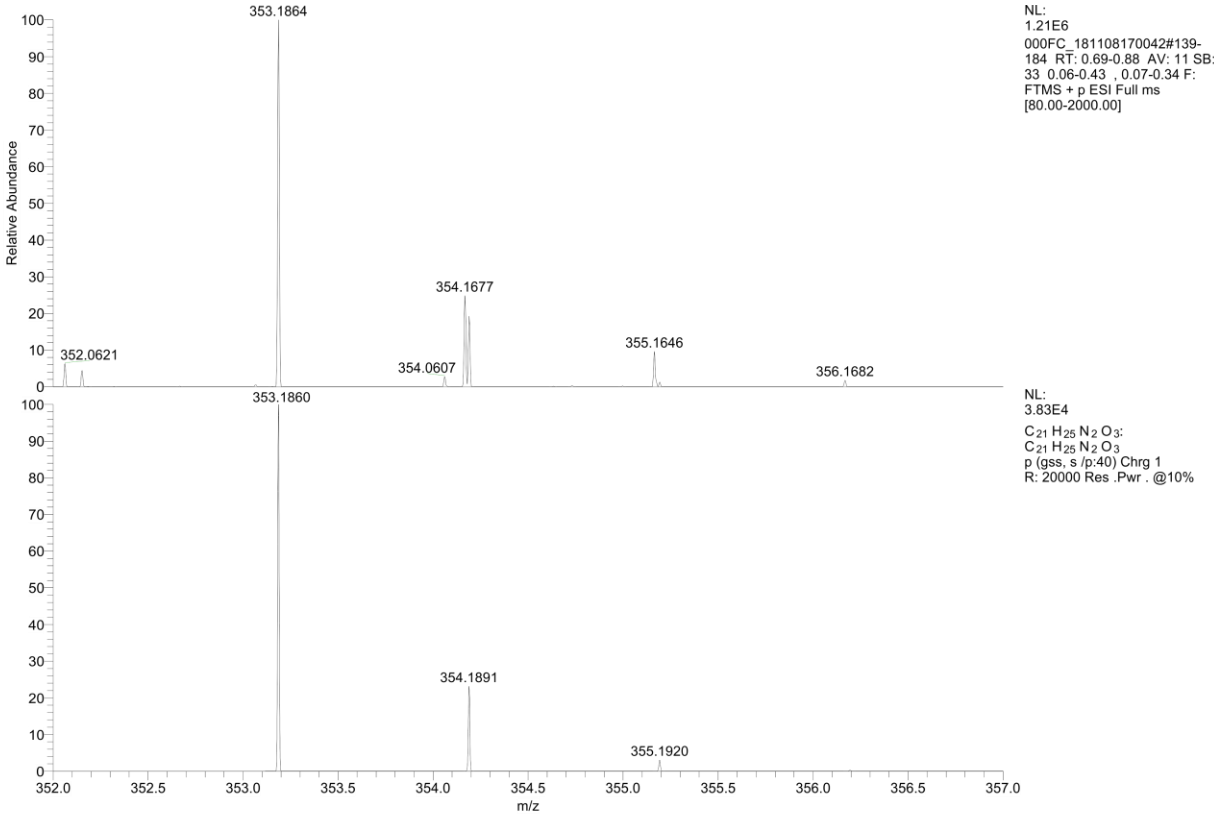

Figure S12 Accurate mass determination with Orbitrap XL of intermediate **I$_3$** ([**I$_3$**]$^+$).

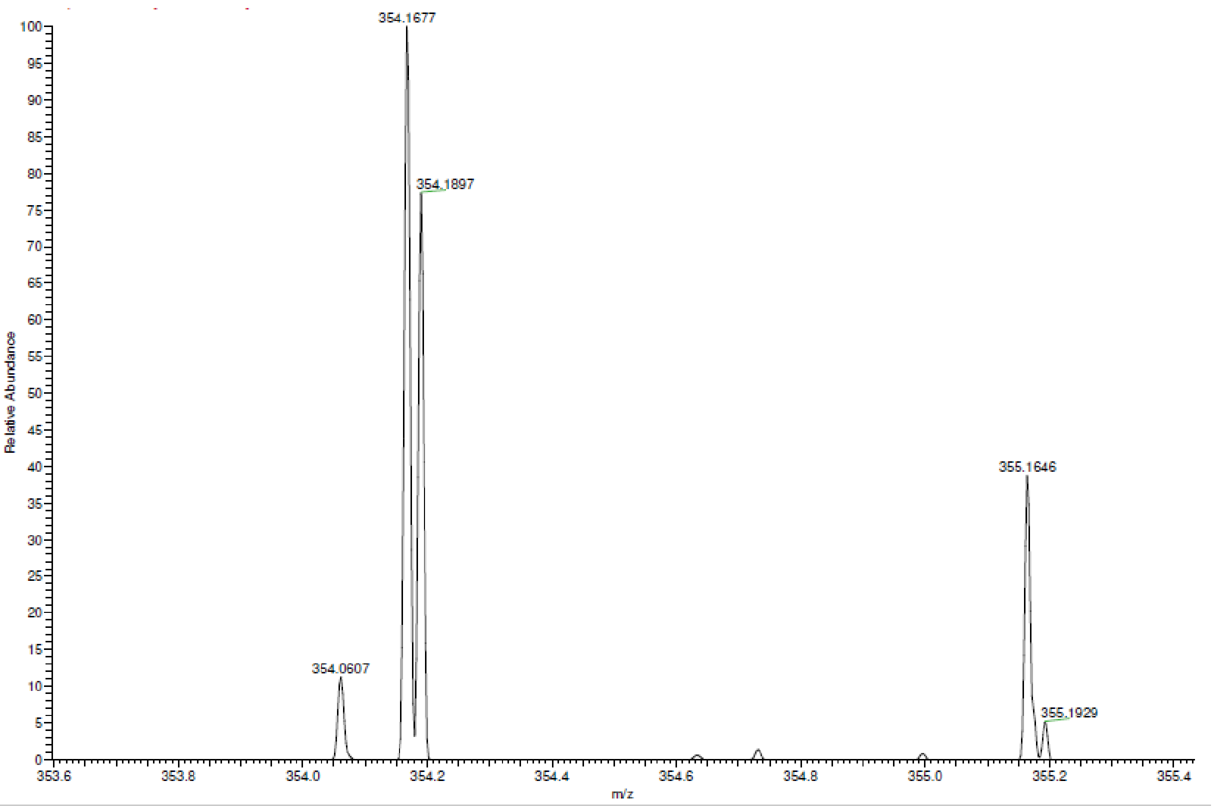

Figure S13 Zoom into ^{13}C peak of **I_3** ([**I_3**]$^+$).

References

[1] List, B.; Hoang, L.; Martin, H. J. *Proc. Natl. Acad. Sci. U. S. A.* **2004,** *101,* 5839–5842. doi:10.1073/pnas.0307979101

[2] Schmid, M. B.; Zeitler, K.; Gschwind, R. M. *Angew. Chem., Int. Ed.* **2010,** *49,* 4997–5003. doi:10.1002/anie.200906629
